# A matter of origin - identification of SEMA3A, BGLAP, SPP1 and PHEX as distinctive molecular features between bone site-specific human osteoblasts on transcription level

**DOI:** 10.3389/fbioe.2022.918866

**Published:** 2022-09-28

**Authors:** Weiping Zhang, Sibylle Rau, Konstantinos Kotzagiorgis, René Rothweiler, Susanne Nahles, Eric Gottwald, Bernd Rolauffs, Thorsten Steinberg, Katja Nelson, Brigitte Altmann

**Affiliations:** ^1^ G.E.R.N Research Center for Tissue Replacement, Regeneration & Neogenesis, Department of Oral- and Craniomaxillofacial Surgery, Medical Center, University of Freiburg, Faculty of Medicine, University of Freiburg, Freiburg, Germany; ^2^ Department of Oral and Craniomaxillofacial Surgery, Medical Center, University of Freiburg, Faculty of Medicine, University of Freiburg, Freiburg, Germany; ^3^ G.E.R.N Research Center for Tissue Replacement, Regeneration & Neogenesis, Department of Prosthetic Dentistry, Medical Center, University of Freiburg, Faculty of Medicine, University of Freiburg, Freiburg, Germany; ^4^ Department of Oral and Maxillofacial Surgery, Berlin Institute of Health, Corporate Member of Freie Universität Berlin, Charité - Universitätsmedizin Berlin, Humboldt-Universität zu Berlin, Berlin, Germany; ^5^ Group 3D Cell Culture Systems, Institute of Functional Interfaces, Karlsruhe Institute of Technology, Karlsruhe, Germany; ^6^ G.E.R.N Research Center for Tissue Replacement, Regeneration & Neogenesis, Department of Orthopedics and Trauma Surgery, Medical Center, University of Freiburg, Faculty of Medicine, University of Freiburg, Freiburg, Germany; ^7^ Department of Oral Biotechnology, Center for Dental Medicine, Medical Center, University of Freiburg, Faculty of Medicine, University of Freiburg, Freiburg, Germany

**Keywords:** bone grafts, 3D-microchip culture, *in vitro* model, gene expression analysis, alveolar bone, iliac crest, osteoblasts, vitamin D

## Abstract

In oral and maxillofacial bone reconstruction, autografts from the iliac crest represent the gold standard due to their superior clinical performance, compared to autografts derived from other extraoral regions. Thus, the aim of our study was to identify putative differences between osteoblasts derived from alveolar (hOB-A) and iliac crest (hOB-IC) bone of the same donor (nine donors) by means of their molecular properties in 2D and 3D culture. We thereby focused on the gene expression of biomarkers involved in osteogenic differentiation, matrix formation and osteoclast modulation. Furthermore, we examined the transcriptional response to Vit.D3 in hOB-A and hOB-IC. Our results revealed different modulation modes of the biomarker expression in osteoblasts, namely cell origin/bone entity-dependent, and culture configuration- and/or time-dependent modulations. SEMA3A, SPP1, BGLAP and PHEX demonstrated the strongest dependence on cell origin. With respect to Vit.D3-effects, BGLAP, SPP1 and ALPL displayed the highest Vit.D3-responsiveness. In this context we demonstrated that the transcriptional Vit.D3-response concerning SPP1 and ALPL in human osteoblasts depended on the cell origin. The results indicate a higher bone remodeling activity of iliac crest than alveolar osteoblasts and support the growing evidence that a high osteoclast activity at the host-/donor bone interface may support graft integration.

## Introduction

In oral and maxillofacial bone reconstruction, autologous bone grafts still represent the gold standard due to their osteogenic (living cells in the graft), osteoinductive (signaling molecules in the graft), osteoconductive (promotion of the revascularization) properties when compared to bone allografts, xenografts and alloplastic substitutes. Common sources of autologous grafts for the regeneration of intraoral bone defects include mandibular and iliac bone blocks, with the latter revealing a superior clinical performance for defects lager than 5 mm (Heberer et al., 2009; Nkenke and Neukam, 2014; Fretwurst et al., 2015a). As a possible cause for this, putative site-specific variations between bone tissue derived from the hip and jaw are discussed. In this context, several studies indicate donor site-specific variations between bone tissues with respect to the bone matrix composition, bone formation and remodeling process (Everts et al., 2009). On a cellular level, earlier work from our group with human bone cells (Wein et al., 2015; Wein et al., 2019) and from other groups using murine and human *in vitro* models (Wan et al., 2016; Kelder et al., 2020) demonstrate that bone-forming osteoblasts derived from skeletal bone have a higher angiogenic and osteoclastogenic potential than osteoblasts derived from the cranial area. These preliminary data together with findings from other groups including bone marrow stem cells (Stefanik et al., 2008; Lee J. T. et al., 2015) corroborate a site-specific molecular and phenotypic heterogeneity of bone cells and thus of bone tissue. As the mechanisms for different graft competence are however still largely unknown, further investigations may identify molecular factors responsible for a superior graft incorporation.

The graft incorporation, particularly in case of cortical autografts, is generally associated with the remodeling of the bone at the graft/host tissue interface that is characterized by a local resorption and new formation of the bone tissue (Roberts and Rosenbaum, 2012; Spin-Neto et al., 2015; Rolvien et al., 2018). Major players in this remodeling process are osteoblasts, osteocytes and recruited and/or resident osteoclasts that exert their activity as bone remodeling units in different anatomical regions of the skeleton. In this process, bone is first resorbed by activated osteoclasts derived from the monocyte/macrophage lineage of the hematopoietic system and afterwards reconstructed by osteoblasts, which subsequently differentiate towards bone matrix-embedded osteocytes (Arias et al., 2018). The resorption and formation of bone tissue within this process are strongly interconnected and orchestrated by paracrine factors (Crockett et al., 2011). Important signaling molecules produced by osteoblasts/osteocytes in this context involve biomarkers 1) of bone matrix homeostasis, such as collagen 1 (COL1A1), osteocalcin (OCN), osteopontin (OPN), alkaline phosphatase (ALPL) and phosphate regulating endopeptidase homolog (PHEX), and 2) osteoclast modulating macrophage colony-stimulating factor (M-CSF), receptor activator of nuclear factor-κB ligand (RANKL), osteoprotegerin (OPG) (Datta et al., 2008; Bellido, 2014), semaphorin 3A (SEMA3A) (Hayashi et al., 2012), ephrinB (EphB) receptors and their ligands (Crockett et al., 2011). Hence, the distribution of osteoclast precursors and the activity of osteoclasts are strongly governed by site-specific osteoblasts and osteocytes. Osteoclast-modulating molecules differently expressed in human site-specific osteoblasts/osteocytes derived from the hip and mandibular bone, and the role they play for the integrative potential of autografts in the regenerative context is to our knowledge not sufficiently known (Wein et al., 2015). As *in vitro* studies point to a differential effect of 1,25D3 on osteoblast response depending on the cell origin and/or phenotype, e.g. different species and cell lines (Van De Peppel and Van Leeuwen, 2014), we further examined the osteoblast response to 1,25D3 with respect to biomarkers involved in osteoclast activation and new bone formation.

Against this background, the aim of the present study was 1) to screen for potential differences in the molecular properties between human osteoblasts derived from alveolar (hOB-A) and matched iliac crest (hOB-IC) bone biopsies and 2) to examine if the transcriptional response to vitamin D varies depending on the donor site. The focus was the comparative gene expression analysis of biomarkers involved in osteogenic differentiation, bone matrix formation and osteoclast modulation.

In order to distinguish between biomarkers modulated in a tissue origin-dependent manner and/or by the *in vitro* culture configuration we used a previously established 3D cell culture model in a microchip-based 3D culture system for osteogenic differentiation of hOB (Altmann et al., 2011; Altmann et al., 2014), together with conventional 2D monolayer cultures. After characterization of the cell culture models, we performed the comparative gene transcription analysis of the hOB-A and hOB-IC in 2D and 3D culture over a period of 21 days, to examine the biomarker expression during osteogenic differentiation (Schiavi et al., 2015; Nasello et al., 2020). The transcriptional response of hOB to 1,25D3 was further examined for a 14 days culture period.

This strategy enabled us to identify differences between bone cells derived from alveolar bone and iliac crest with respect to their osteogenic and osteoclastogenic potential on molecular level, and provided new information on the differential 1,25D3 responsiveness of site-specific human osteoblasts. The distinct gene expression pattern in hOB-IC thereby points to a higher capacity of hOB-IC to foster osteoclast genesis and/or activity and osteogenic differentiation when compared with hOB-A. Our results further revealed that the biomarker expression in hOB depended on the 1) cell origin, 2) culture configuration (2D *versus* 3D) and/or culture time, and 3) on vitamin D treatment. Interestingly, the expression of some biomarkers with very low expression level in hOB-A in 2D could be upregulated by using hOB in 3D culture. Altogether, the results of this study provide new insights into the molecular basis associated with site-specific properties of human bone tissue, and thus contribute to a biological-clinical (re-)orientation of innovative therapeutic approaches in the field of oral bone augmentation surgery and bone tissue engineering in general.

## Materials and methods

### Isolation and cultivation of primary human osteoblasts

Primary osteoblasts were prepared from human alveolar bone and iliac crest bone explants of the same donor (healthy female donors; mean age 61.7 ± 5.2 years, *n* = 9). The bone samples were harvested during iliac onlay bone grafting as described earlier (Fretwurst et al., 2015b). The collection of the bone samples and usage of the primary osteoblasts for scientific purposes was approved by the Ethics Committee of the Albert-Ludwigs-University, Freiburg, Germany (vote Nr. 603/15) and Charité Berlin (vote Nr. EA4/049/13), and informed consent was given by the patient. Research was performed in accordance with relevant guidelines and regulations. The bone samples were cleaned in phosphate-buffered saline (PBS) to remove residues of blood and soft and/or bone marrow tissue. In order to ensure sterility of the bone explant cultures, the cleaned bone specimens were additionally sterilized in an iodide solution for 30s, subsequently washed with PBS and transferred to Petri dishes. This technique has proven successful to prevent a possible contamination of the bone explants with bacteria from the oral cavity during harvesting and at the same time to enable the outgrowth of osteoblastic cells from the explants. The bone explants, and later the isolated cells, were cultured in expansion medium (EM) at 37°C with 5% CO2. The EM consisted of Dulbecco’s Modified Eagle’s Medium (DMEM, Life Technologies) supplemented with 10% (v/v) fetal bovine serum (Sigma-Aldrich), 2% (v/v) glutamine (Life Technologies) and 0.1 mg/ml kanamycin (Sigma-Aldrich). The primary osteoblasts were obtained by spontaneous outgrowth of the cells from the bone explants. To gain a sufficient amount of cells for the experiments including the characterization of the *in vitro* cell models and the comparative gene expression analysis, the isolated cells were propagated under established culture conditions. In detail, cells were passaged after reaching 70–80% confluence using 0.05%Trypsin/0.02% EDT in PBS. In order to avoid extensive *in vitro* aging and phenotypic changes of the cells, all experiments were carried out with osteoblasts of early passages 4 and 5 (Yang et al., 2018). This passage numbers have proven to maintain donor site-/patient-specific cell characteristics across the *in vitro* cultivation (Wein et al., 2015; Wein et al., 2019; Kelder et al., 2020). Extracellular matrix mineralization was induced by incubating confluent 2D monolayer (ML) and 3D-microchip cultures with osteogenic medium (OM) consisting of EM supplemented with 10 mM β-glycerophosphate disodium salt hydrate (Sigma-Aldrich), and 50 μg/ml sodium l-ascorbate (Sigma-Aldrich). In order to examine the gene transcription after vitamin D treatment, osteoblasts were cultured in OM supplemented with 10^−8^ M 1,25-dihydroxyvitamin D3 (1,25D3, Sigma-Aldrich, D1530) for 7 and 14 days.

### 3D-microchip culture

The chip scaffolds (300MICRONS, Karlsruhe, Germany) used in this study consisted of a porous polycarbonate membrane (10 µm thickness, 3 µm pore diameter) with cylindrical microcvities (300 µm diameter and 250 µm depth) arranged in an array of 25 × 25 cavities (625 cavities in total) (Giselbrecht et al., 2006). The generation of 3D-microchip cultures was performed as previously described (Altmann et al., 2014). In detail, the scaffolds were first treated with an alcohol series of ethanol, including 100, 70, 50 and 30%, to exhaust the air from the microcavities, washed with sterile water and finally washed with PBS. In order to improve initial cell adhesion inside the microcavities the microstuctured area of the chip scaffolds was coated with 0.1% human fibronectin (FN) solution (Sigma-Aldrich). For a better comparability of the 3D-microchip and 2D monolayer (ML) cultures, Petri dishes and multiwell plates for 2D ML were also coated with the same concentration of FN. The 3D-microchips were inoculated with 0.8 × 10^6^ cells in 150 μL EM for 2 h at 37°C and 5% CO_2_ in an incubator. Due to the geometry of the chip array and the FN coating of the cavities, this resulted in the formation of 625 adherent multicellular aggregates with a uniform size. The 3D chips were then transferred in 6-well cell culture plates and covered with 3 ml EM. After 24 h the medium was changed to OM.

### Evaluation of cell proliferation

The proliferation capacity of the isolated cells was analyzed by means of metabolic activity and DNA concentration in 2D monolayer (ML) cultures as previously described (Altmann et al., 2017). For this, 5 × 10^3^ cells/ml/well in expansion medium (EM) were seeded in 24-well-plates (corresponds to 2.6 × 10^3^ cells per cm^2^) in triplicates and examined at days 1, 3 and 7. The metabolic activity was determined in the culture medium by the alamarBlue (AB) assay (Invitrogen) according to the manufacturer’s instructions and quantified by measuring fluorescence (PerkinElmer, EnSight). The percentage of AB reduction in the samples was calculated using a 100% reduced AB control as reference, which was produced according to the manufacturer´s protocol. The DNA quantification was performed with specimens previously used for the AB assay. After AB sampling the attached cells were therefore washed twice with PBS and lysed by a freeze-thaw cycle at −80°C in 250 μL TE buffer (Invitrogen) (Ng et al., 2005; Altmann et al., 2013). The TE buffer was thereby directly added to the attached monolayer after the PBS wash step. The DNA concentration was determined by the PicoGreen dsDNA assay kit (Thermo Fischer).

### Histological analysis

Osteoblast arrangement and aggregate formation in the 3D-microchips were visualized under basic culture conditions (EM) at days 7, 21 and 28 in resin-embedded sections. The 3D cultures were fixed with 4% formaldehyde in PBS and embedded in Technovit 8100 (Heraeus Kulzer). Serial sagittal sections of 7 µm (Leica 2065 Supercut slicer) were stained with hematoxylin-eosin (HE). In order to examine the mineralization potential and thus osteoblastic phenotype of the isolated cells under 2D and 3D culture conditions, the cells were cultured in OM for 28 days. Calcium deposition into the extracellular matrix was visualized by Van-Kossa staining of formaldehyde-fixed 2D ML (basic cell characterization) or sagittal sections of 3D aggregates. Sagital sections of 3D-microchip cultures was performed as described above.

### Scanning electron microscopy (SEM) and energy-dispersive X-ray spectroscopy (EDX)

To analyze cell morphology and the nature of the matrix mineralization in the osteogenic 3D-microchip cultures, we performed scanning electron microscopy (SEM) for morphology analysis and energy-dispersive X-ray spectroscopy (EDX) to detect calcium (Ca) and phosphorus (P) (given as atom mass%) (JEOL, JSM-IT100) in the samples. The Ca/P ratio gives information on the presence of hydroxyapatite. The cells were cultured as described above in OM for 7 and 28 days, fixed with 4% formaldehyde, dehydrated in ascending alcohol series 70, 80, 90, 100% for 1 h each, critical point dried (CPD 030 Critical Point Dryer, Bal-Tec AG, Balzers), and sputter coated with gold palladium at 40 mA for 80 s (SCD 050, Balzers).

### Cell viability

The cell viability in 2D ML and 3D-microchip cultures was analyzed by fluorescence-based live/dead staining at days 7 and 28 in OM. The cells were incubated with 5 µM Syto16 (Invitrogen) and 1 µM propidium iodide (PI) (Sigma-Aldrich) in EM at 37°C for 30 min. The stained samples were immediately imaged by Zeiss Observer.Z1 microscope. The quantitative analysis of the live/dead cells ratio was performed with the FIJI 2.1.0/1.53c software.

### Gene expression analysis

The gene transcription analysis of the biomarkers listed in Table 1 in 2D ML and 3D-microchip cultures (duplicates) was performed by droplet digital PCR (ddPCR). Total mRNA was isolated at days 7, 14 and 21 (QIAGEN RNeasy Plus Micro kit, Qiagen) and analyzed by Nanodrop (Thermo Scientific) and capillary electrophoresis (Highsens RNA analysis kit, Experion Automated Electrophoresis System, Bio-Rad). Reverse transcription was performed with the Advantage RT-for-PCR Kit (Takara) according to the manufacturer´s protocol. Droplet digital PCR was carried out with the QX200 System from Bio-Rad in 20 µL final volume containing specific ddPCR Gene Expression Probe Assays (Supplementary Table S2) and cDNA equivalent to 0.5–2 ng initial RNA. Since variation in ddPCR assays can result from intrinsic properties of individual RNA samples or may be introduced during the experimental steps preceding the ddPCR assays (Zmienko et al., 2015), we performed a duplex ddPCR using our genes of interest (FAM fluorophore) together with a reference gene (HEX fluorophore). The reference gene represented the direct internal control of an individual reaction (Taylor et al., 2017; Dmiqe-Group and Huggett, 2020). In order to choose a stable reference gene for our experimental set up, we examined the transcription levels of hypoxanthine phosphoribosyltransferase 1 (HPRT1) and hydroxymethylbilane synthase (HMBS), which were demonstrated to have a stable expression during the osteogenic differentiation in osteoblasts under different culture conditions and at different culture time (Stephens et al., 2011). Based on our evaluation we used HPRT1 as reference gene for the normalization of the absolute copy numbers of the genes of interest (GOI) because it was stably expressed in an appropriate concentration/copy number in the different culture configurations (2D and 3D) and over time (Supplementary Figure S4A). The normalization of the GOI to HPRT1 reduced the variance between the technical replicates or more precisely between individual ddPCR reactions (SupplementaryFigures S4B,C).

**TABLE 1:** Target genes and reference gene for the comparative gene transcription analysis in osteoblasts derived from alveolar and iliac crest bone (Bonewald, 2011; Stephens et al., 2011; Bellido, 2014). Primer Assay information are listed in Supplementary Table S2.

Gene	Description	Expressed in	Function
RUNX2	Runt related transcription factor 2	Osteoblasts	Osteogenic differentiation
PDPN	Podoplanin	Early osteocytes	Formation of dendrites
PHEX	Phosphate regulating endopeptidase homolog, X-linked	Early and mature osteocytes	Phosphate metabolism
COL1A1	Collagen type 1 alpha 1 chain	Osteoblasts	Bone matrix formation
BGLAP	Bone gamma-carboxyglutamate protein	Osteoblasts	Mineralization
SPP1	Secreted phosphoprotein 1	Osteoblasts Osteocytes	Mineralization; adhesion of osteoclasts to mineralized ECM
ALPL	Alkaline phosphatase liver/bone/kidney	Osteoblasts	Mineralization
OPG	TNF receptor superfamily member 11b	Osteoblasts, Osteocytes	Inhibitor of osteoclastogenesis
CSF1	Colony stimulating factor 1	Osteoblasts, osteocytes	Proliferation and survival of osteoclasts and osteoclast precursor cells
EPHB4	EPH receptor B4	Osteoblasts	Inhibits osteoclast activity; stimulates osteogenic differentiation
SEMA3A	Semaphorin 3A	Osteoblasts	Inhibits osteoclastogenesis; stimulates osteogenic differentiation
VDR	Vitmin D receptor	Osteoblasts Osteocytes	Positive regulator of bone resorption; facilitates Ca-absorption
HPRT1	Hypoxanthine phosphoribosyltransferase 1	Osteoblasts Osteocytes	Purine synthesis through the purine salvage pathway

### Statistical analysis

The Shapiro-Wilk test was used to test if the data were normally distributed. For not normally distributed data we used the Wilcoxon signed rank test, and for normally distributed data we used the Paired *t*-test to identify significant differences between the test groups. The statistical data analysis and visualization was performed with the *Python* libraries Seaborn, SciPy and OriginPro 2021b. With respect to the sample size, we used cells from nine donors for the gene expression analysis. Since the available cell amount for the present study was limited, for some biomarkers the donor size was less than nine. The sample size used for the statistical analysis is indicated in the figure captions. If samples were excluded for technical reasons, a repeat of the experiments was not possible. In this case the paired statistical analysis was performed with the minimal available donor number.

## Results

### Validation of the osteogenic in vitro models derived from alveolar and iliac crest bone of the same donor

#### Proliferation capacity of cells derived from alveolar and iliac crest bone in 2D monolayer culture conditions

Since former work demonstrated differential proliferation behavior between hOB-A and hOB-IC derived from three donors (Wein et al., 2015), we first examined the proliferation capacity of the bone-derived cells. The proliferation was determined based on the metabolic activity and DNA concentration in 2D monolayer (ML) cultures at days 1, 3, 7 and 14 in expansion medium. A longer evaluation period was not reasonable because the cells reached confluence after 2 weeks culture and didn´t proliferate further, likely due to the high cell density-based contact inhibition. This was the reason why the cell proliferation analysis was restricted to 2D ML cultures and did not include high-density 3D aggregate cultures.

**FIGURE 1:**
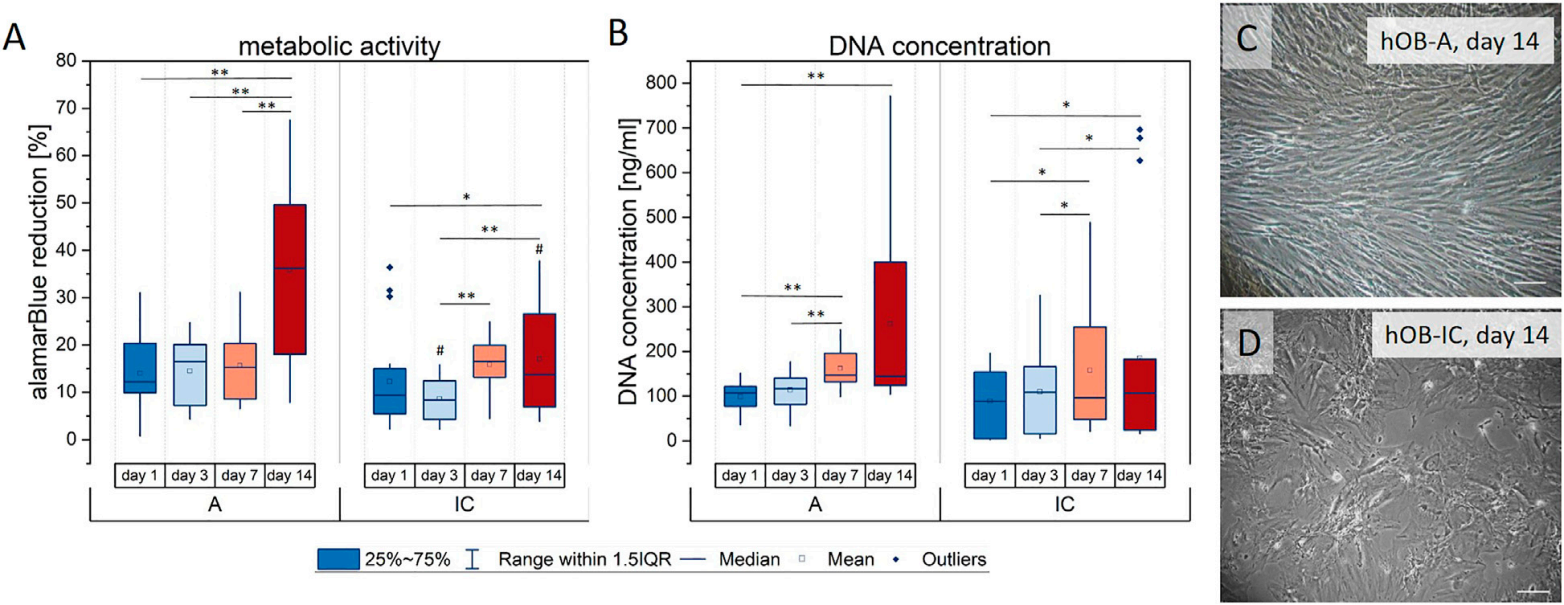
Proliferation of human osteoblasts derived from alveolar bone (hOB-A) and matched iliac crest (hOB-IC) of the same donor (6 donors) in expansion medium. The proliferation capacity was determined by **(A)** the metabolic activity given by the reduction of the reporter dye alamarBlue in the mitochondrial respiratory chain (metabolic activity is given as percentage of a 100% reduced control) and **(B)** the DNA concentration that is proportional to the cell number at days 1, 3, 7 and 14. **(C,D)** Bright field micrograph of hOB-A and hOB-IC after 14 days 2D-monolayer culture. *p < 0.05 and **p < 0.01 for comparison between culture days; ^#^ *p* < 0.05 for comparison between hOB-A and hOB-IC at the corresponding time point; Wilcoxon signed-rank test, *n* = 6. Scale bars: 100 µm.

The results presented in Figure 1 demonstrate that the metabolic activity increased until day 14 in both cell types, whereby hOB-IC cultures had a lower alamarBlue reduction rate at days 3 and 14, when compared to hOB-A (Figure 1A). The increase in the metabolic activity over culture time further coincided with rising DNA amount until day 14 in hOB-A and hOB-IC which was in a similar scale for both cell types (Figure 1B). These data indicate that iliac crest-derived cells had a lower metabolic activity and thus proliferation rate than corresponding hOB-A. This assumption was supported by bright field microscopy images of 14-days old cultures which further demonstrated that hOB-IC did not reach complete confluence but formed a spread morphology when compared with alveolar bone cells (compare Figures 1C,D). Such a proliferation trend has already been observed for iliac crest-derived cells in former work (Wein et al., 2015). The osteogenic phenotype of the isolated cells was verified by the matrix mineralization potential in long-term cultures (28 days) in osteogenic culture conditions (Supplementary Figure S1).

#### Aggregate formation and matrix mineralization in 3D-microchip culture

**FIGURE 2:**
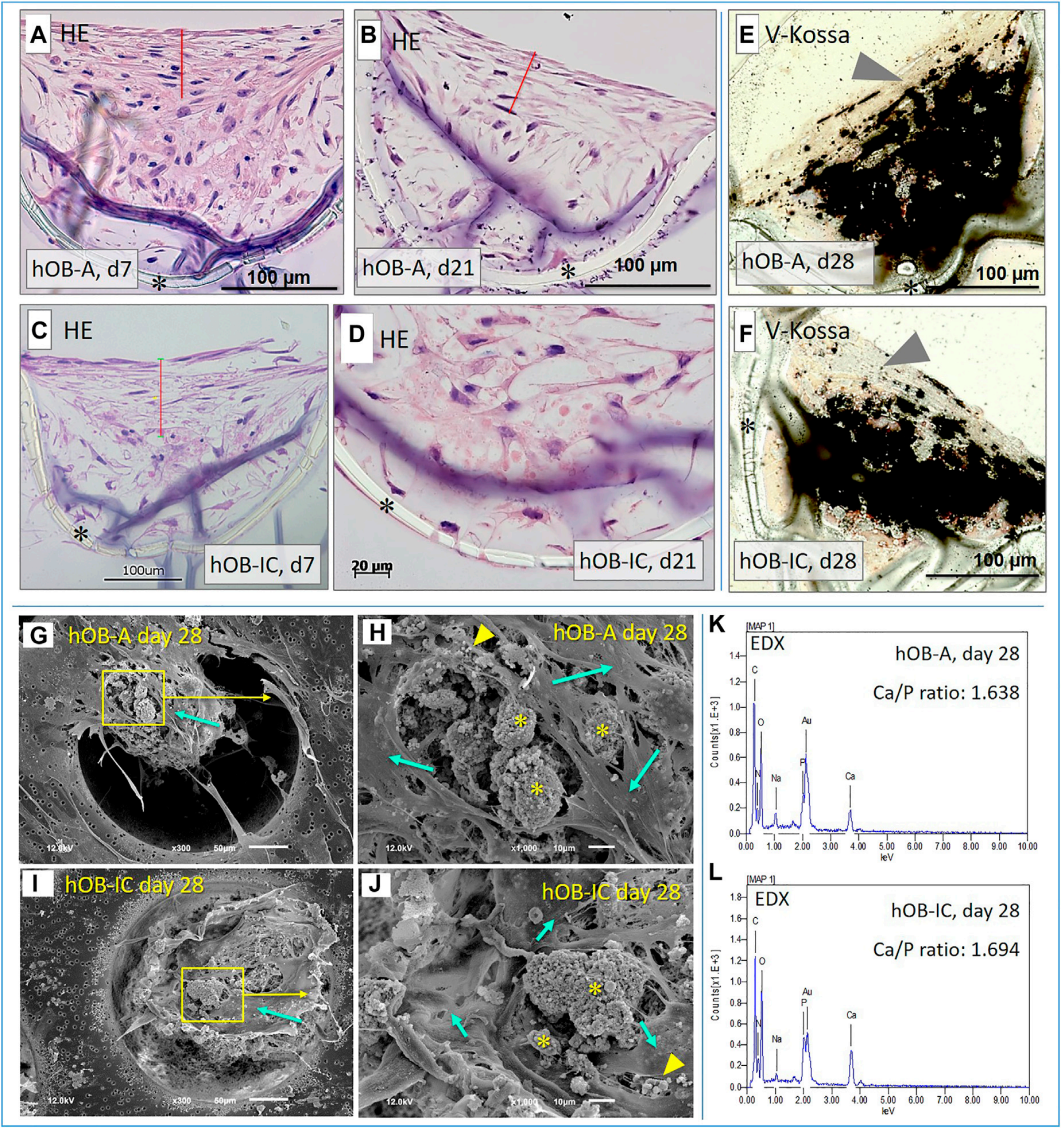
Evaluation of the cell aggregation and matrix mineralization in 3D-microchip cultures. **(A−D)** Representative images of sagittal resin-embedded sections of hOB in 3D-microchip culture stained with hematoxylin and eosin. The cells were cultured in basic culture conditions (expansion medium) and analyzed at days 7 and 21. Red line indicates the horizontally oriented cell layer at the opening of the cavity. Image in **(D)** shows a high magnification of the central area in the cavity. **(E,F)** Representative images of sagittal resin-embedded sections of hOB in 3D-microchip culture stained with the Van Kossa method. The cells were cultured for 28 days in 3D-microchips under osteogenic conditions to ensure a significant mineralization of the extracellular matrix. Black areas in the center of the cavities visualize the calcium deposition and thus areas of mineralization. Arrow heads point to the upper non-mineralized cell layer at the opening of the cavity. **(G−J)** Representative SEM micrographs of osteoblasts derived from alveolar (hOB-A) and iliac crest bone (hOB-IC) cultured for 28 days in a 3D-microchip device under osteogenic culture conditions. Stars = Mineralization nodules, arrowheads = mineralization vesicles, arrows = cells. **(K,L)** Representative EDX spectra of hOB-A and hOB-IC cultures after 28 days culture under osteogenic conditions. *Porous membrane of the microchip. Scale bars in **(G,I)**: 50 μm; scale bars in **(H,J)**: 10 µm.

After having established the bone cell cultures, we examined the aggregate formation of the cells in the 3D-microchip device. For this, the cells were seeded into the microchips and cultured over a period of 21 days in basic culture conditions. Cell arrangement in the cavities of the chips was visualized by hematoxylin and eosin stain (HE) of resin-embedded sections. The histological analysis of the sections revealed that hOB formed multicellular aggregates inside the cavities with differential orientation and morphology depending on their localization in the cavities. In detail, cells in the apical area, at the opening of the cavity, were aligned horizontally with elongated morphology (Figures 2A–C–C, area marked with a red line), whereas cells inside the cavity were randomly distributed with a more spread morphology (Figure 2D, bottom of the chip is marked with a star). This situation was observed after 7 and 21 days of culture, suggesting that cell morphogenesis in the microchips was completed as early as 7 days. The horizontally oriented cell layer was, irrespective of the culture time points, approximately 86 ± 11 µm thick (marked as red line in Figures 2A–C). Regarding the matrix mineralization in long-term 3D cultures under osteogenic conditions, demonstrated by Van Kossa staining, it is noticeable that the calcium deposition (black areas in Figures 2E,F) was mainly detectable in the aggregate center while the previously described horizontally orientated cell layer persisted in a non-mineralized state at the apical side (Figures 2E,F, arrow head).

To scrutinize the cell morphology and the nature of the matrix mineralization in the osteogenic hOB-microchip cultures, we next performed scanning electron microscopy (SEM) and energy-dispersive X-ray spectroscopy (EDX) with 28 days old cultures under osteogenic conditions. As shown in Figures 2G–J, structures resembling large mineralization nodules (Figures 2H,J, stars) and small mineralization vesicles (Figures 2H,J, yellow arrowheads) were visible under the top cell layer of the aggregates (Figures 2G–J), mint green arrows). The chemical analysis of these structures by EDX revealed that these structures were composed of 15.31 (±6.90) mass% calcium (Ca) for hOB-A and 12.77 (±2.98) mass% for hOB-IC, and 8.46 (±3.73) mass% phosphorus (P) for hOB-A and 7.45 (±1.57) for hOB-IC (see also Supplementary Table S1). Of note was here that at day 7 (Supplementary Table S1) values for Ca and P were lower in hOB-A, namely 8.23 %mass ±4.40 for Ca and 5.18 %mass ±2.49, when compared to corresponding hOB-IC, i.e., 15.52 %mass± 2.98 for Ca and 8.33 %mass ±1.43 for P. Hence, Ca and P %mass values in hOB-IC cultures were similar at day 7 and 28, whereas similar Ca %mass values in hOB-A where only reached at day 28. This observation points to an earlier Ca deposition in hOB-IC *versus* hOB-A and deserves further study. The Ca/P ratio was 1.75 (±0.22) for hOB-A and 1.71 (±0.09) for hOB-IC cultures. As a ratio in excess of 1.6 indicates hydroxyapatite (Sotiropoulou et al., 2015), the round structures observed by SEM imaging were mineralization nodules composed of hydroxyapatite. Overall, the results from the histological staining, SEM and EDX analysis demonstrate that hOB derived from alveolar and iliac crest bone formed multicellular aggregates with a high mineralization potential under osteogenic 3D culture conditions.

#### Cell viability in 2D monolayer and 3D-microchip culture

**FIGURE 3:**
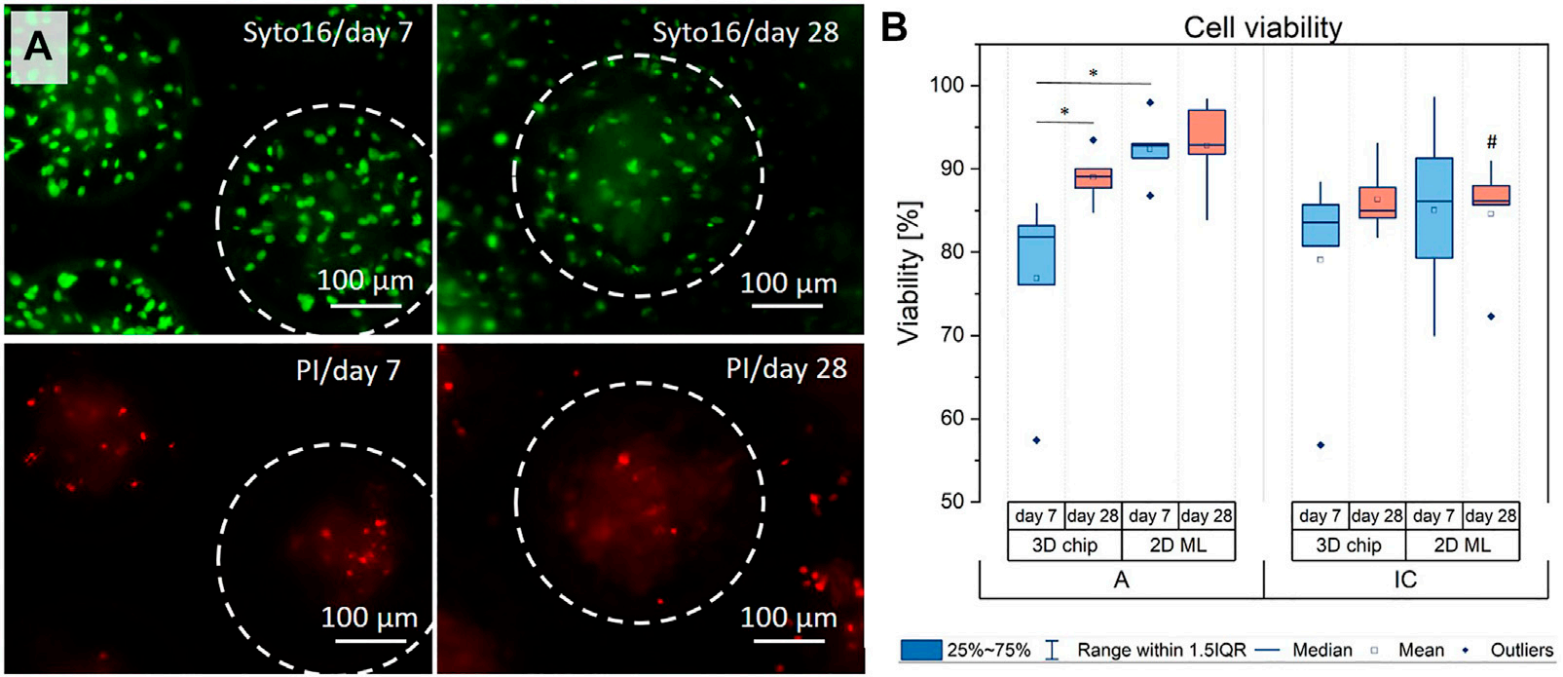
**(A)** Representative images of the live/dead staining of osteoblasts derived from alveolar bone (hOB-A) at days 7 and 28 in 3D-microchips under osteogenic conditions. The corresponding fluorescence images of hOB-IC in 3D-microchips, and 2D monolayers of both cell types are provided in Figures S2 and S3. **(B)** For quantitative analysis of the viability fluorescence images were analyzed by FIJI 2.1.0/1.53c software. Dotted circles indicate cell aggregates in one single microchip cavity. Syto16 (green fluorescence): living cells; PI (red fluorescence): dead cells. *p < 0.05 for comparison between culture types and time points; ^#^ *p* < 0.05 for comparison between hOB-A with hOB-IC at the corresponding time points and culture modes. Paired *t*-test, *n* = 5.

The results from the histological and SEM/EDX analysis point to putative oxygen, nutrient and/or metabolite gradients inside the multicellular hOB aggregates under basic culture conditions, which coincided with a strong ECM mineralization in the center of the aggregate under osteogenic conditions. To test for adverse effects of the high-density 3D culture conditions, we analyzed the cell viability in 3D culture and compared it with corresponding 2D ML cells. As cell culture evaluation included early and long-term cultures, we analyzed the cell viability at days 7 and 28 by fluorescence-based live/dead staining with Syto16 (living cells) and propidium iodide (PI, dead cells). The qualitative analysis revealed that in all culture configurations, i.e., 2D vs 3D, hOB-A vs hOB-IC, PI-positive cells were scattered detectable. Larger PI-positive areas inside the center of the cavities/aggregates were not observed, excluding necrotic cell death (Figure 3A). The software-based quantification of the live/dead staining confirmed these observations and demonstrated that cell viability in 3D cultures was 76.88% (±11.44) and 79.08% (±12.75) for hOB-A and hOB-IC at day 7, and 89.00% (±3.19) and 86.36% (±4.31) for hOB-A and hOB-IC at day 28 (Figure 3B). The viability values for the corresponding 2D monolayer cultures were slightly higher than in 3D cultures (statistically not significant with exception of hOB-A, day 7) with values of 92.38% (±4.00) and 85.07 (±10.99) for hOB-A and hOB-IC at day 7, and 92.81% (±5.69) and 84.62 (±7.18) for hOB-A and hOB-IC at day 28. With respect to the 28 days long-term 2D cultures, our data further revealed that the cell viability of hOB-IC was significantly lower than in matched hOB-A cells (Figure 3B). These findings suggest a sufficient oxygen and nutrient supply of the cells inside the microchip cavities and thus in the 3D culture model, and that hOB-IC viability decreased in long-term cultures when compared to hOB-A.

### Transcriptional analysis of biomarkers involved in osteogenic differentiation, bone matrix formation and osteoclast modulation in osteoblasts derived from alveolar and iliac crest bone

#### Identification of biomarkers expressed in a cell origin-dependent manner (alveolar *versus* iliac crest bone)

**FIGURE 4:**
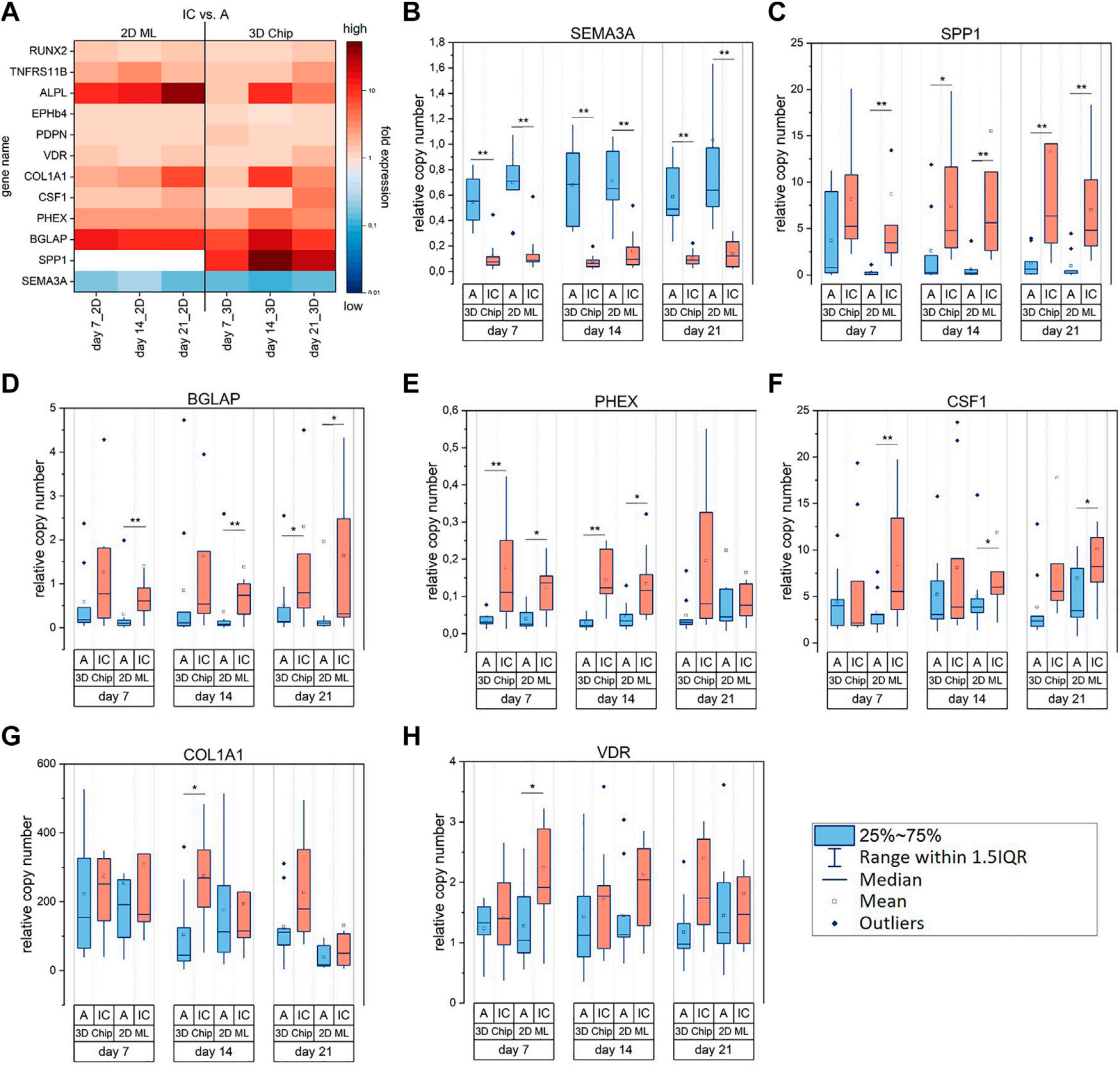
Relative gene expression of biomarkers differently expressed between osteoblasts derived from alveolar bone (hOB-A, blue) and iliac crest (hOB-IC, red) of the same donor. hOB were cultured in 3D-microchip and 2D ML culture under osteogenic conditions. **(A)** The transcriptional difference between hOB-IC and hOB-A is given by the fold expression, i.e., hOB-IC/hOB-A ratio of normalized copy numbers and describes the fold expression of biomarkers in hOB-IC *versus* hOB-A. A ratio of 1 means no difference, >1 higher expression, and <1 lower expression. **(B–H)** Relative transcript levels in each cell type for biomarkers which were significantly different between hOB-IC and hOB-A. The copy numbers of the target genes at days 7, 14 and 21 were normalized to copy numbers of the reference gene HPRT1 (hypoxanthine phosphoribosyl-transferase 1). The relative copy numbers of each experimental group, i.e. cell type, were compared and tested for statistically significant difference. *p < 0.05 **p < 0.01 for comparison of hOB-A with hOB-IC; Wilcoxon signed-rank test, n = 9 donors. SEMA3A (semaphorin-3A), SPP1 (osteopontin), BGLAP (osteocalcin), PHEX (phosphate regulating endopeptidase, X-linked), CSF1 (colony stimulating factor 1), COL1A1 (collagen type 1 alpha 1 chain) and VDR (vitamin D receptor). White areas in the heatmap **(A)** indicate out of range values for SPP1 in 2D ML with values higher than 160-fold expression.

After demonstrating the suitability of our cell culture model for the osteogenic *in vitro* differentiation of cells derived from alveolar and iliac crest bone, we next performed the comparative gene transcription analysis at days 7, 14 and 21. As initially mentioned, our results from this transcription analysis revealed different modulation modes of the biomarkers in hOB under study. In this section, we focus on biomarkers primarily expressed as a function of cell origin, i.e. biomarkers differently expressed between hOB-A and hOB-IC. Figure 4A gives an overview of the transcriptional differences between hOB-IC *versus* hOB-A for all examined biomarkers, displayed as fold expression, and Figures 4B–H present the relative transcript levels in each cell type for biomarkers which were significantly different between hOB-IC and hOB-A. Biomarkers which were not expressed significantly different between hOB-IC and hOB-A are presented in Supplementary Figure S5. Of the 12 biomarkers under study listed in Table 1, transcription of SEMA3A (semaphorin-3A), SPP1 (osteopontin), BGLAP (osteocalcin) and PHEX (phosphate-regulating gene with homologies to endopeptidases on the X chromosome) demonstrated the strongest dependence on the cell origin (Figure 4A). Even though the data indicated that ALPL (alkaline phosphatase) was also stronger expressed in hOB-IC vs hOB-A (Figure 4A), detailed statistical analysis did not reveal a significant difference in the ALPL transcription levels between these 2 cell types (Supplementary Figure S5; 3D chip_day7: *p* = 0.64, 2D ML_day7: *p* = 0.25, 3D chip_day14: *p* = 0.054, 2D ML_day14: *p* = 0.30, 3D chip_day21: *p* = 0.054, 2D ML_day21: *p* = 0.25).

As presented in Figures 4B–E significant different expression levels of aforementioned biomarkers in hOB-A and hOB-IC were identified in six (SEMA3A), five (SPP1) and four (BGLAP, PHEX) culture conditions out of six. Culture conditions describe here the culture system (3D chip and 2D ML) and culture time (days 7, 14, 21). SEMA3A was the only gene among the four biomarkers which was expressed higher in hOB-A than in matched hOB-IC (Figure 4B), whereas SPP1, BGLAP and PHEX were expressed higher in hOB-IC (Figures 4C–E). Another remarkable result was the significant up-regulation of SPP1 in hOB-A in 3D culture conditions at day 7 when compared to the corresponding 2D ML cultures. In this case interesting because SPP1 had generally very low transcription levels in hOB-A under conventional 2D ML conditions and in comparison to matched hOB-IC (Figure 4C).

Further biomarkers displaying higher expression levels in hOB-IC with matched hOB-A were CSF1 (colony stimulating factor 1; Figure 4F), COL1A1 (collagen type 1 alpha 1 chain; Figure 4G) and VDR (vitamin D receptor; Figure 4H). However, we point out that a significant differential expression between hOB-A and hOB-IC was only detectable in three (CSF1) and one (COL1A1, VDR) out of six culture conditions. In detail, CSF1 expression was higher in hOB-IC than in corresponding hOB-A cultures exclusively under 2D culture conditions (Figure 4F), whereas COL1A1 was up-regulated under 3D culture conditions at day 14 in hOB-IC with matched hOB-A (Figure 4G), and VDR showed higher expression levels in hOB-IC in 2D ML at day 7 *versus* hOB-A (Figure 4H).

Taken together, these data demonstrate that among the group of analyzed genes involved in osteoclast modulation and ECM formation, SEMA3A, SPP1, BGLAP and PHEX represented significant distinctive features between hOB-A and hOB-IC across the conditions tested.

#### Identification of biomarkers expressed in a cell culture system (2D *versus* 3D)- and/or time-dependent manner

In addition to cell origin-specific gene transcription, we identified further biomarkers exclusively modulated 1) by the cell culture technique, i.e., 2D ML *versus* 3D-microchip culture, and 2) by the culture time. Biomarkers that were not modulated significantly by the culture technique and/or time are presented in Supplementary Figure S6.

**FIGURE 5:**
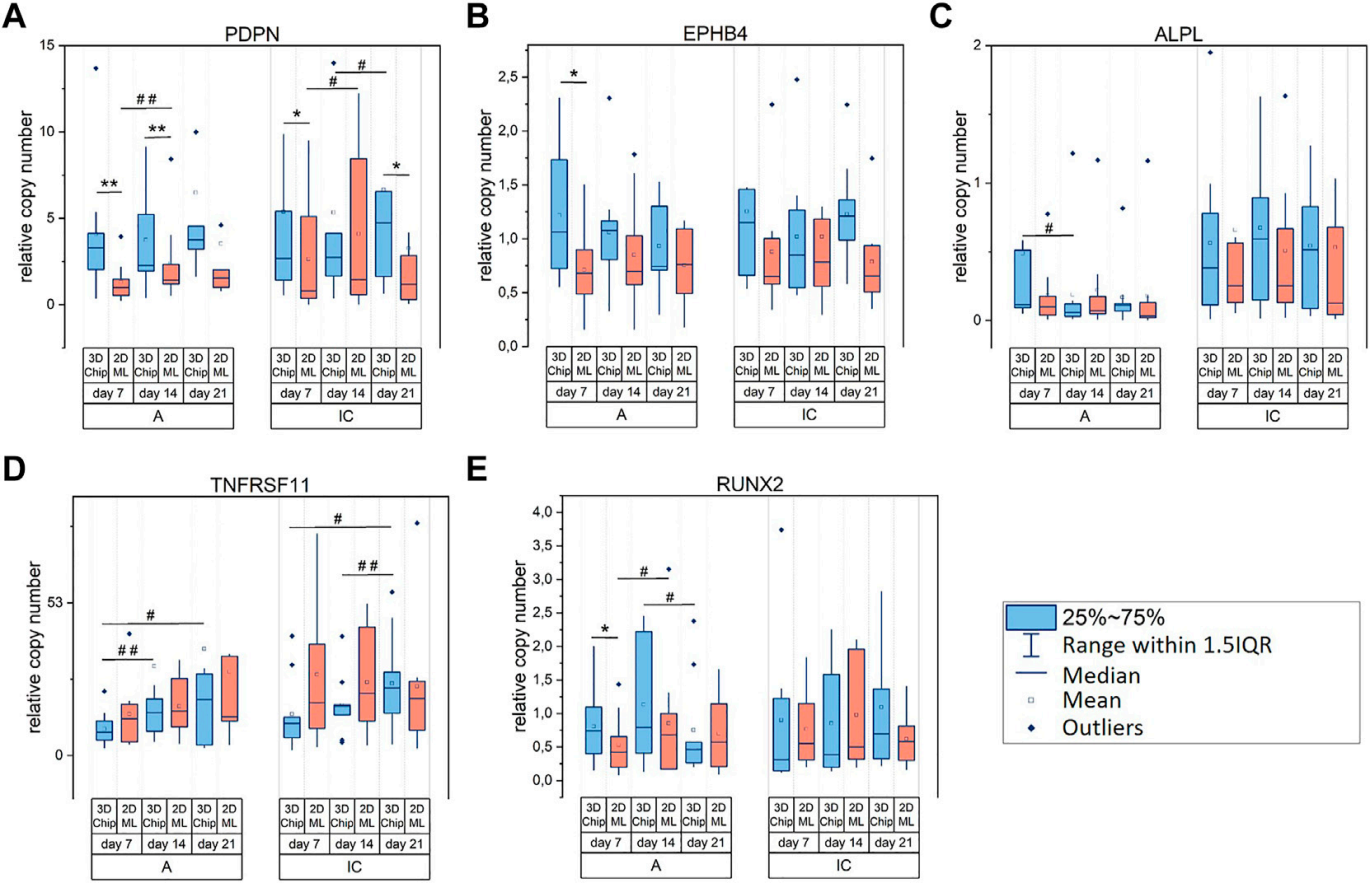
**(A–E)** Relative gene expression of biomarkers in osteoblasts modulated by cell culture configuration (2D *versus* 3D) and/or culture time. Color code of the comparison groups: 3D-microchip in blue and 2D ML in red. Osteoblasts derived from alveolar bone (hOB-A) and iliac crest (hOB-IC) of the same donor were cultured in 3D-microchip and 2D ML culture under osteogenic conditions. The copy numbers of target genes at days 7, 14 and 21 were normalized to copy numbers of the reference gene HPRT1 (hypoxanthine phosphoribosyl-transferase 1). *p < 0.05, **p < 0.01 for comparison of 3D-microchipwith 2D ML culture; ^#^ *p* < 0.05, ^##^ *p* < 0.01 for comparison of culture time; Wilcoxon signed-rank test, *n* = 9 donors. PDPN (podoplanin), EPHB4 (ephrin receptor B4), ALPL (alkaline phosphatase, liver/bone/kidney), TNFRSF11B (TNF receptor superfamily member 11b) and RUNX2 (runt related transcription factor 2).

With respect to biomarkers modulated by the cell culture technique, our analysis revealed that PDPN encoding for podoplanin, which is produced in early osteocytes during dendrite formation (Ikpegbu et al., 2018), had higher transcription levels in 3D culture compared to matched 2D ML in both cells types, i.e., hOB-A and hOB-IC (Figure 5A). This result indicated that the 3D microenvironment in the microchips supported the dendrite formation in hOB when compared to 2D ML. Further biomarkers showing increased gene transcription in 3D *versus* 2D were EPHB4/ephrin receptor B4 in hOB-A (Figure 5B) and RUNX2/runt related transcription factor 2 in hOB-A (Figure 5E) at day 7. In addition to culture configuration-dependent modulation, PDPN was further regulated in a time-dependent manner showing a weak but significant up-regulation during culture time in hOB-A/2D ML and hOB-IC in 3D-microchip and 2D ML culture (Figure 5A).

Genes exclusively regulated over culture time were TNFRSF11B (Figure 5D) which encodes for tumor necrosis factor receptor superfamily member 11B, also known as osteoprotegerin, and ALPL/alkaline phosphatase, liver/bone/kidney (Figure 5C). Importantly, TNFRSF11B transcription was exclusively regulated in 3D-microchip cultures showing thereby an up-regulation during the whole culture period in both cell types, i.e., hOB-A and hOB-IC (Figure 5D).

Overall, these data suggested that PDPN/podoplanin was mainly modulated by the spatial arrangement of the cellular microenvironment, thereby showing a significant up-regulation in 3D culture with matched 2D ML in hOB-A and hOB-C. In turn, TNFRSF11B was exclusively modulated in 3D culture in a time-dependent manner in both cell types when compared to 2D ML.

#### Identification of biomarkers modulated by vitamin D

Our gene transcription analysis data in Figure 4H and the information from other *in vitro* studies on human, murine and rat osteoblasts (Van De Peppel and Van Leeuwen, 2014) indicate a differential osteoblast responsiveness to 1,25D3 that may depend on the phenotype and/or cell origin. In order to identify potential differences in the transcriptional response of hOB-A and hOB-IC to 1,25D3 we next examined the gene transcription profile in hOB-A and hOB-IC in 1,25D3-treated and untreated cultures. According to our own preliminary tests and reports by other groups (Wijenayaka et al., 2015; Posa et al., 2016), we cultured hOB-A and hOB-IC in osteogenic culture conditions supplemented with 10^−8^ M 1,25D3 for 7 and 14 days, and analyzed the gene transcription of the biomarkers under study (Table 1) at the given time points.

**FIGURE 6:**
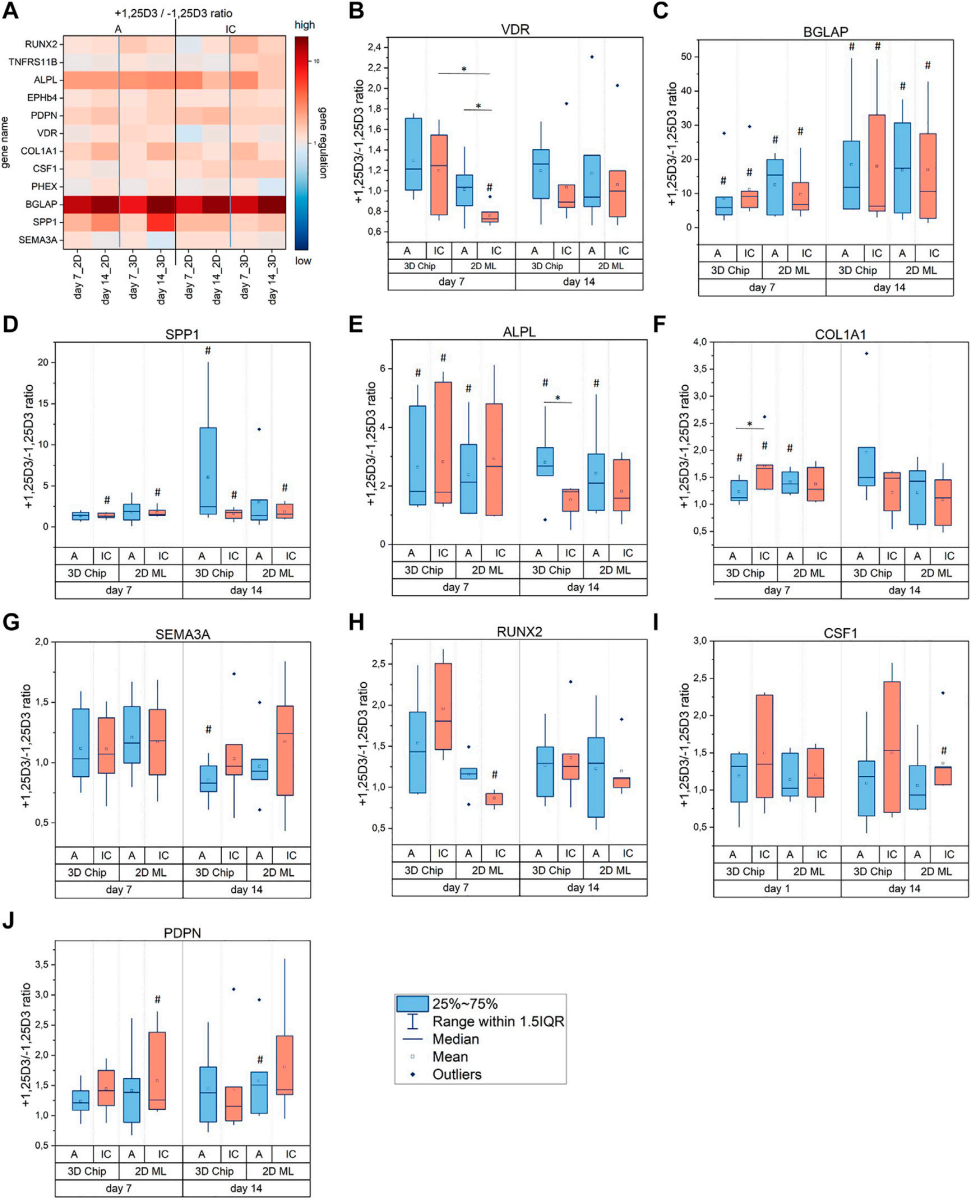
**(A–J)** Effect of 1,25D3 treatment on gene expression in osteoblasts derived from alveolar bone (hOB-A, blue) and iliac crest (hOB-IC, red). The cells were cultured in 3D-microchip and 2D ML culture under osteogenic conditions for 7 and 14 days with or without (+/-) addition of 1,25D3 (1,25-dihydroxyvitamin D3). The copy numbers of the target genes were normalized to copy numbers of the reference gene HPRT1 (hypoxanthine phosphoribosyl-transferase 1). The effect of 1,25D3 treatment is given by the +1,25D3/-1,25D3 ratio of normalized copy numbers and describes the fold expression of biomarkers in 1,25D3-treated *versus* untreated cell cultures. A ratio of 1 means no regulation, >1 up-regulation, and <1 down-regulation. ^#^ *p* < 0.05 for comparison of 1,25D3 treated with non-treated cultures (+1,25D3 vs. -1,25D3); *p < 0.05 for comparison of the 1,25D3 effect between culture modes, i.e. culture configuration, time and cell origin; Wilcoxon signed-rank test, *n* = 7 donors; *n* = 5 donors for COL1A1.

We identified VDR, BGLAP, SPP1, ALPL, COL1A1, SEMA3A, RUNX2, CSF1 and PDPN as target genes that were regulated by 1,25D3 in hOB-A and/or hOB-IC. As illustrated in Figure 6A, BGLAP, SPP1 and ALPL showed the strongest responsiveness to 1,25D3 since they were significantly up-regulated in all culture configurations (2D and 3D) and at all culture time points (Figures 6C–E). The modulation mode followed thereby a specific pattern. In detail, BGLAP was up-regulated in both cell types with rising trend from day 7 to day 14 (Figure 6C), whereas SPP1 was mainly modulated in hOB-IC (Figure 6D) and ALPL in hOB-A (Figure 6E). Interestingly, SPP1 was also significantly up-regulated in 1,25D3-treated hOB-A under 3D culture conditions at day 14, whereas 1,25D3 did not show an effect on SPP1 transcription in the corresponding 2D ML.

The effect of 1,25D3 on the other biomarkers, namely VDR, COL1A1, SEMA3A, RUNX2, CSF1 and PDPN was less pronounced than on aforementioned genes. Different transcription levels were detectable in only one (VDR, SEMA3A, RUNX2, CSF1), two (PDPN) or three (COL1A1) culture conditions. Of note is that VDR, SEMA3A and RUNX2 were the only genes, which were down-regulated after 1,25D3 treatment (Figures 6B,G,H). VDR and RUNX2 down-regulation coincided thereby in hOB-IC under 2D ML culture conditions at day 7, whereas SEMA3A was significantly down-regulated in hOB-A in 3D culture at day 14. Biomarkers that were not modulated by 1,25D3 included TNFRSF11B, EPHB4 and PHEX (Supplementary Figure S7).

Our findings revealed that among the highly 1,25D3-responsive genes, SPP1 and ALPL modulation by 1,25D3 was related to the osteoblast phenotype. Furthermore, SPP1 modulation in hOB-A was exclusively detectable in 1,25D3-treated 3D cultures.

## Discussion

Since clinical and *in vitro* data suggest site-specific molecular and phenotypic heterogeneity of bone forming cells, i.e. osteoblasts, in autologous bone grafts, we isolated primary human osteoblasts (hOB) from the alveolar and matched iliac crest bone and compared their gene transcription of biomarkers involved in bone regeneration. In order to identify a cell origin-dependent expression of biomarkers, we used a previously established 3D cell culture model in a microchip-based 3D culture system for the osteogenic differentiation of hOB (Altmann et al., 2011; Altmann et al., 2014), and additionally conventional 2D monolayer cultures. In order to obtain reliable data on cell origin-dependent phenotype, we performed all experiments, including initial cell characterization and subsequent transcription analysis, with cells derived from the same donors (9 donors in total). This approach required the propagation of the isolated cells by subcultivation up to passage 4–5. The application of subcultured osteoblasts with low passage numbers was reasonable since the cells kept their proliferative capacity and ECM mineralization potential up to passage 5. Hence, cell senescence at this early passage numbers could be excluded (Yang et al., 2018). With respect to tissue origin-specific cell characteristics, former own and other work demonstrated that donor-site specific as well as patient-specific variations are maintained on molecular level during *in vitro* cell culture at passage 4 and 5 (Wein et al., 2015; Aurich et al., 2018; Wein et al., 2019; Kelder et al., 2020).

The validation of our osteogenic 3D *in vitro* model demonstrated that hOB formed irrespective of the bone entity multicellular aggregates with high mineralization capacity and viability over a period of 28 days under osteogenic conditions. With respect to cell morphogenesis, cells located in the aggregate center differed in terms of morphology and spatial orientation from cells in the apical layer at the opening of the cavity, whose thickness corresponded to the oxygen diffusion range found in native tissue (Helmlinger et al., 1997). This indicates that the observed differential morphogenesis was due to a possible oxygen concentration gradient. It is indeed conceivable that a mass transport gradient, concerning oxygen, nutrients and metabolites, forms between the opening and bottom of the cavities, and thus generates small distinct microenvironments inside the cavities, which give rise to distinct cell morphologies. Other reports describing such mass transport gradients in 3D cell culture models support this assumption (Lee M. K. et al., 2015; Figueiredo et al., 2018; Magliaro et al., 2019) Interestingly, the ECM mineralization process and thus the *in vitro* cell differentiation appeared to be favored by the microenvironment present in the central cavity/aggregate areas. One possible explanation for this observation might be the aforementioned mass transport gradients between the opening and bottom of the cavities, and/or a possible accumulation of secreted mineralization vesicles inside the aggregates. Such mass transport gradients and mild hypoxic areas are generally present in static 3D cultures as well as in native tissue, and play a regulatory role during new bone formation and mineralization *in vivo* (Rouwkema et al., 2010; Sammarco et al., 2014). With respect to oxygen gradients, *in vitro* studies demonstrated that ECM mineralization and osteogenic differentiation is favored under hypoxic conditions (Hirao et al., 2007; An and Heo, 2018). However, if such putative low oxygen concentration in the cavity and/or aggregate center may be the causative for this observed mineralization can only be answered in future work.

After verifying the osteogenic phenotype of the isolated cells, i.e. human osteoblasts, and the suitability of our 3D cell culture model for the osteogenic *in vitro* differentiation of hOB, we performed the comparative gene transcription analysis with hOB-A (alveolar bone) and hOB-IC (iliac crest bone). As mentioned earlier, for this we used a 3D-microchip and 2D ML cultures to distinguish between biomarkers expressed depending on the cell origin and/or on the *in vitro* culture technique. With this strategy we identified SEMA3A (semaphorin-3A), SPP1 (osteopontin), BGLAP (osteocalcin) and PHEX (phosphate-regulating gene with homologies to endopeptidases on the X chromosome) as distinctive features between alveolar bone and iliac crest on transcription level. SEMA3A/semaphorin-3A was the only gene showing a higher transcription rate in hOB-A than in hOB-IC, whereas the later three genes were expressed stronger in hOB-IC with matched hOB-A. Among these biomarkers different transcription rates between hOB-IC *versus* hOB-A were so far only reported for SPP1/osteopontin. The higher SPP1 expression in our hOB-IC cultures is thereby in accordance with an earlier work of our group (Wein et al., 2019) and with results presented by Kelder et al. (Kelder et al., 2020), which used human alveolar and knee bone osteoblasts. In light of these data, these concordant results further substantiate the reliability and reproducibility of the applied human osteoblast-based *in vitro* models in the present and aforementioned studies.

With respect to the function of the identified biomarkers, it is striking that all four genes encode proteins, which play an important role in osteoclast and osteoblast modulation, as well as in regulating bone matrix mineralization. In detail, SEMA3A/semaphorin-3A inhibits mature osteoclast differentiation of precursor cells and at the same time stimulates the osteogenic differentiation in osteoblasts and bone marrow mesenchymal stem cells through the Wnt/β-catenin signaling pathway (Hayashi et al., 2012). In the context of bone graft integration, it is conceivable that a lower SEMA3A transcription in iliac crest graft-osteoblasts *versus* mandibular graft-osteoblasts may better support the differentiation of local osteoclast precursors, and thus the presence of mature osteoclasts, at the host-/donor bone interface.

A further protein with dual function in the regenerative context is SPP1/osteopontin that promotes osteoclastogenesis and osteoclast activity, and negatively regulates hydroxyapatite crystal growth during ECM mineralization (reviewed in (Singh et al., 2018)). Osteopontin is present throughout the bone, were it binds to apatite crystals, and more importantly, accumulates preferentially at matrix-matrix interfaces, e.g. at cement lines between old and new bone, healing bone surfaces and where bone mineralization is initiated, and at cell-matrix interfaces (quiescent and activated bone surfaces, lacunae and canaliculi) (Mckee and Nanci, 1996). In this context, several studies suggest that osteopontin in bone surfaces interfacing with cells mediates the attachment of osteoclasts to bone and stimulates their migration (Standal et al., 2004). Closely associated with osteopontin is the phosphate-regulating neutral endopeptidase PHEX which cleaves full-length osteopontin, including its mineralization-inhibiting peptide motive. The complete degradation of full-length osteopontin by PHEX prevents the accumulation of mineralization-inhibiting peptides and by this contributes to the local control of crystal growth in bone mineralization (Barros et al., 2013). Hence, the higher transcription rates of SPP1 and PHEX in hOB-IC *versus* hOB-A in the present study point to a PHEX-OPN co-expression in hOB, which may be important to maintain the local equilibrium between osteoclastogenesis and ECM mineralization. Neves et al. (2016) which demonstrated a PHEX-OPN co-expression and PHEX-mediated regulation of OPN function in squamous carcinoma cells support this assumption.

BGLAP/osteocalcin is synthesized by osteoblasts and represents the most abundant non-collagenous matrix protein in bone. The protein is crucial for the alignment of apatite crystals parallel to collagen fibers and by this plays a crucial role for the bone strength (Komori, 2019; Manolagas, 2020; Moriishi et al., 2020). In bone healing, osteocalcin appears to induce an earlier onset and increased rate of bone remodeling during bone healing, and thus may accelerate bone regeneration. This assumption was stated by Rammelt et al. (Rammelt et al., 2005), who demonstrated that bone formation and osteoclast recruitment around osteoclacin-modified implants occurred earlier in a rat *in vivo* model when compared to the control implants. Other research groups describing a chemotactic activity of osteocalcin on osteoclasts and osteoclast-like cells *in vitro* and *in vivo* (Glowacki and Lian, 1987; Chenu et al., 1994) further support such osteoclast recruiting effect of osteocalcin.

Taken together, these results indicate that the distinct gene expression pattern detected in hOB-IC points to a higher capacity of hOB-IC 1) to foster osteoclast genesis and/or activity, 2) to promote osteogenic differentiation and 3) to support the bone strength in new bone, when compared with hOB-A. This points to an overall higher activity of iliac crest graft-osteoblasts in terms of a bone remodeling process, and by this supports the growing evidence that a high osteoclast activity at the host-/donor bone interface supports graft integration (Rolvien et al., 2018). In order to substantiate our results on transcriptional level, we will next focus on the bone-site specific protein expression of the identified biomarkers.

In addition to the cell origin-dependent biomarkers, we further identified genes that were primarily modulated by the culture configuration (2D *versus* 3D) and/or culture time. These included PDPN/podoplanin, EPHB4/ephrinB4-receptor und RUNX2/runt related transcription factor 2 and TNFRSF11B/TNF receptor superfamily member 11b (also known as osteoprotegerin). PDPN transcription was thereby up-regulated in both cell types, i.e., hOB-A and hOB-IC, under 3D culture conditions when compared with matched 2D ML. This observation is in line with results from (Boukhechba et al., 2009) who demonstrated that PDPN is up-regulated in human osteoblasts during osteocytic differentiation in 3D culture when compared with 2D ML.

EPHB4 and RUNX2, likewise, were expressed higher in 3D *versus* 2D, albeit to a lower extent, namely in only one of six culture conditions. It is noteworthy, that the expression of EPHB4, which has a similar bidirectional signaling function on osteoblasts and osteoclasts as SEMA3A/semaphorin-3A (Crockett et al., 2011), was not expressed in a cell origin-dependent manner. This result substantiates the unique position of SEMA3A as a molecular distinctive feature of hOB-IC. With respect to RUNX2, we observed in addition to the higher transcription in 3D culture a weak time-dependent modulation. As RUNX2 is an essential transcription factor for osteogenic differentiation that is regulated in a stringent chronological sequence during osteoblast maturation (Maruyama et al., 2007; Qin et al., 2019), the low and/or stable constitutive transcription level in our hOB cells may be due to their mature phenotype. This is supported by the observation that RUNX2 is down-regulated during osteoblast maturation in native bone tissue (Maruyama et al., 2007).

Another interesting result was the significant up-regulation of SPP1 in hOB-A in 3D culture conditions at day 7, when compared to the corresponding 2D ML cultures, because SPP1 had generally very low transcription levels in hOB-A under conventional 2D ML conditions and in comparison to matched hOB-IC. The latter is in line with our previous work using 2D ML demonstrating a significant lower SPP1 expression in hOB-A at day 7 with matched hOB-IC in multiple donors (Wein et al., 2019). In the present study, however, the up-regulation of SPP1 in hOB-A under 3D culture conditions abolished the different SPP1 expression pattern between hOB-A and hOB-IC at this specific time point. This observation underlines the impact of the cell culture technique for the modulation of the cell behavior *in vitro*. Hence, the inclusion of 3D cell culture models in bone cell research and biomarker screening may be helpful to identify those biomarkers that are also sensitive to the spatial arrangement of the *in vitro* microenvironment.

With respect to the vitamin D effect on the biomarkers under study, we identified VDR, BGLAP, SPP1, ALPL, COL1A1, SEMA3A, RUNX2, CSF1 and PDPN as 1,25D3-responsive genes in hOB. Of them, SPP1 and ALPL appeared to be modulated by 1,25D3 in a cell type specific manner. In detail, SPP1 was up-regulated in hOB-IC and ALPL in hOB-A when compared to untreated controls in all culture conditions, i.e. in 2D and 3D, as well as at all time points. By contrast, BGLAP was up-regulated in both cell types in all culture conditions by 1,25D3. The gene expression modulation of SPP1, ALPL and BGLAP by vitamin D3 in osteoblasts is well-documented (reviewed in (Van De Peppel and Van Leeuwen, 2014)) and thus substantiates the results gained with our hOB *in vitro* model in the present study. However, a cell type-specific modulation of SPP1 and ALPL in hOB-A and hOB-IC has not been demonstrated so far. Our finding is supported in part by Kelder et al. (Kelder et al., 2020) who demonstrated an up-regulation of SPP1 in osteoblasts from long bone *versus* alveolar bone at day 21 after vitamin D treatment. Furthermore, our data demonstrated that SPP1 was also up-regulated by 1,25D3 in hOB-A in one single culture condition, namely in 3D culture at day 14. This is remarkable, because SPP1 up-regulation in hOB-A in the present study was exclusively detectable in 1,25D3-treated and untreated 3D cultures, but not in 2D ML cultures. Hence, SPP1 appears to take a special position among the examined biomarkers as its transcription depended on 1) the cell origin (hOB-A vs hOB-IC), 2) the spatial arrangement of the microenvironment (2D vs 3D) and 3) the presence of vitamin D.

We further found that RUNX, VDR and SEMA3A were the only genes, which were down-regulated by 1,25D3 in hOB – albeit the down-regulation reached only in one culture condition statistical significance. It is noteworthy, that RUNX2 and VDR were simultaneously down-regulated in hOB-IC at one specific time point and culture mode, namely at day 7 in 2D ML. This observation suggests a co-expression of both genes under this culture condition, and is supported by other reports indicating a functional cooperation between RUNX2 and VDR in the regulation of osteogenic gene transcription (Paredes et al., 2004; Shen and Christakos, 2005). In contrast, SEMA3A was significantly down-regulated in 1,25D3-treated hOB-A under 3D culture conditions at day 14, and thus identifies SEMA3A as 1,25D target gene in human osteoblasts. Up to now, vitamin D responsiveness has only been demonstrated for SEMA3B in a human osteoblast cell line and in primary murine osteoblasts (Sutton et al., 2008).

In conclusion, the results of our comparative in vitro study on transcriptional level point to a putative higher activity of iliac crest osteoblasts versus matched alveolar bone osteoblasts in terms of the bone remodelling process, and by this, supports the growing evidence that a high osteoclast activity at the host-/donor bone interface supports bone graft integration. Furthermore, we demonstrated that the cell’s origin, i.e., the respective bone entity, was not only decisive for constitutive biomarker expression, but also for the transcriptional response of vitamin D target genes in human osteoblasts. Hence, treatment concepts including the targeted modulation of specific biomarkers during bone graft and/or biomaterial integration into the host tissue by local application or release of signalling molecules, e.g., vitamin D, may in the future consider the origin of the involved bone entities. With regard to the latter, the choice and/or concentration of the signalling molecules, such as vitamin D, could be adjusted to the target bone type. With respect to clinical practice, the knowledge gained by the present work contributes to an evidence-based clinical decision-making on the choice of the transplant source to maximize the clinical performance in individual cases.

In the context of our results, it should be kept in mind, however, that the findings of this study have to be seen in light of some limitations. First, our study focused on female donors with a mean age of approximately 61 years. As there may exist gender and age specific differences in the gene expression of the biomarkers under study, the presented data may be only in part generalizable. Despite this fact, our results point out that further research on this issue has the potential to gain new clinically relevant insights into bone biology of autografts. A second limitation of the study is certainly the fact that we could not provide the total donor number for each experimental approach. As with majority of *in vitro* studies working with primary human cells, the limited cell yield from tissue specimen and/or limited number of available human tissue specimen are challenging for the experimental design and practical realization. Further miniaturization of *in vitro* cell culture scaffolds and devices, as well as highly sensitive analysis methods on single cell-level may help to overcome this limitation in the future.

## Data Availability

The raw data supporting the conclusions of this article will be made available by the authors, without undue reservation.
