# Supplementary material for: A matter of origin - identification of SEMA3A, BGLAP, SPP1 and PHEX as distinctive molecular features between bone site-specific human osteoblasts on transcription level
Source: Front Bioeng Biotechnol. 2022 Sep 28;10:918866. doi: 10.3389/fbioe.2022.918866 (PMC9554416; doi:10.3389/fbioe.2022.918866)
Supplement: Supplementary file 1 [file DataSheet1.docx]

Supplementary Material

# Van Kossa staining of osteoblastic 2D monolayer cultures

**
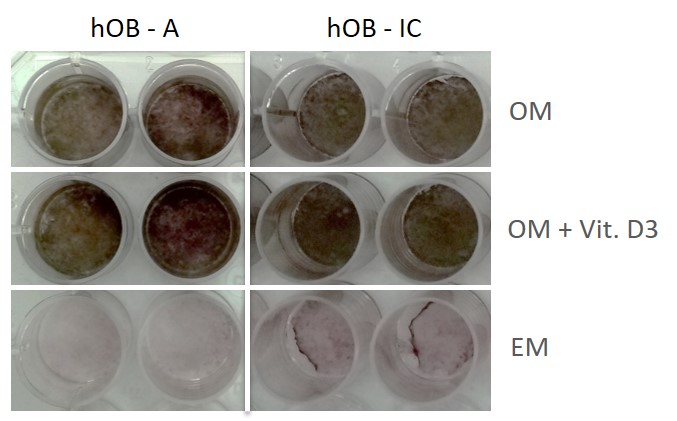
**

**Supplementary Figure 1.** Von Kossa staining of 2D monolayer cultures derived from alveolar (hOB-A) and iliac crest (hOB-IC) bone for detecting calcium deposition into the extracellular matrix and by this verifying the osteogenic phenotype of the cells. The cells were cultured for 28 days in osteogenic medium (OM) and osteogenic medium supplemented with 1,25-dihydroxyvitamin D3 (OM + 1,25D3) to trigger extracellular matrix mineralization, and in expansion medium (EM) resulting in no mineralization (negative control). Black areas visualize the calcium deposition and thus areas of mineralization.

## 2 Calcium and phosphorus content of matrix vesicles

**Supplementary Table S1.** Element analysis of 3D-microchip cultures with respect to calcium (Ca) and phosphorus (P) by EDX. Mean ± SD (n= 4 - 8)

| **Cell type** | **Day** | **Ca [mass%]** | **P [mass%]** | **Ca [mass%] /**  **P [mass%]** |
| --- | --- | --- | --- | --- |
| hOB-A | 7 | 8.23 ± 4.40 | 5.18 ± 2.49 | 1.72 ± 0.54 |
|  | 28 | 15.31 ± 6.90 | 8.46 ± 3.73 | 1.75 ± 0.22 |
| hOB-IC | 7 | 15.52 ± 2.98 | 8.33 ± 1.43 | 1.86 ± 0.07 |
|  | 28 | 12.77 ± 2.98 | 7.45 ± 1.57 | 1.71 ± 0.09 |

# 3 Live/dead staining of osteoblasts derived from iliac crest bone in 3D

**
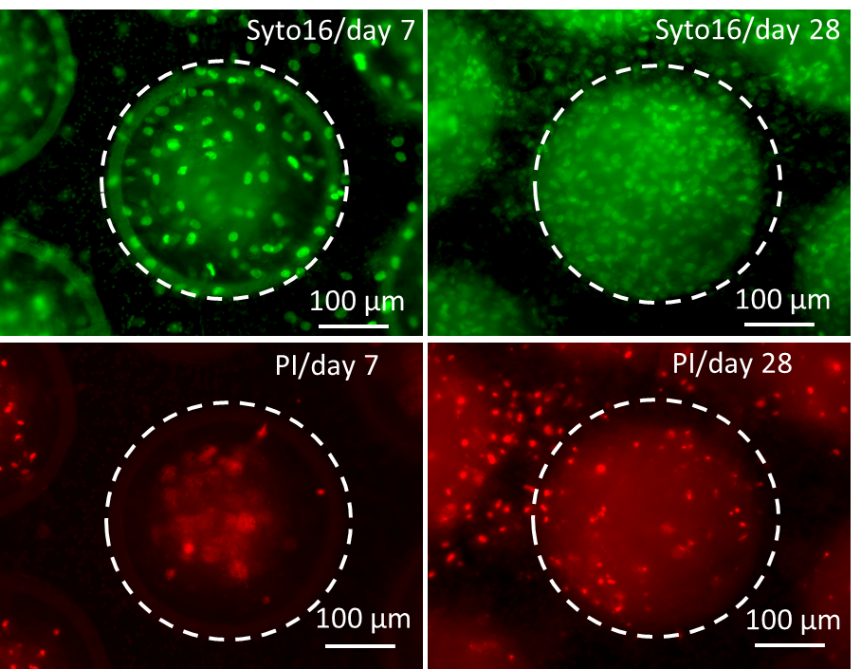
**

**Supplementary Figure S2.** Representative images of the live/dead staining of osteoblasts derived from iliac crest bone (hOB-IC) at days 7 and 28 in 3D-microchips. Dotted circles indicate cell aggregates in one single microchip cavity. Syto16 (green fluorescence): living cells; PI (red fluorescence): dead cells.

# 4 Live/dead staining of osteoblasts derived from iliac crest and alveolar bone in 2D


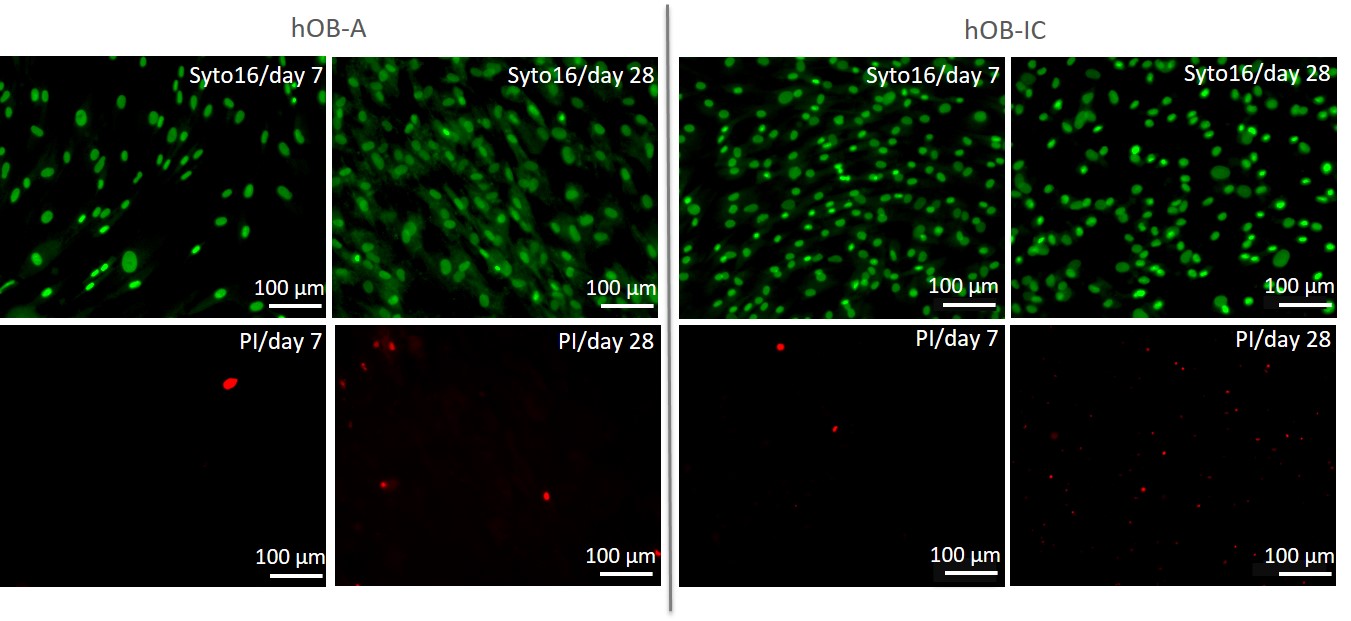


**Supplementary Figure S3.** Representative images of the live/dead staining of osteoblasts derived from alveolar (hOB-A) and iliac crest (hOB-IC) bone at days 7 and 28 in 2D monolayer culture. Syto16 (green fluorescence): living cells, PI (red fluorescence): dead cells. Syto16 (green fluorescence): living cells; PI (red fluorescence): dead cells.

## 5 PCR primer sequences of analyzed biomarkers

**Supplementary Table S2.** ddPCR gene expression assays/primer homo sapiens from Bio-Rad

| **Gene** | **Assay ID** | **Chemistry** | **Probe**  **Fluorophore** | **Amplicon length** | **MIQE Context** |
| --- | --- | --- | --- | --- | --- |
| RUNX2 | dHsaCPE5035834 | Probe | FAM | 83 | hg19\|chr6:45459738-45479990:+  TAGGGCGCATTCCTCATCCCAGTATGAGAGTAGGTGTCCCGCCTCAGAACCCACGGCCCTCCCTGAACTCTGCACCAAGTCCTTTTAATCCACAAGGACAGAGTCAGATTACAGACCCCAGG |
| PDPN | dHsaCPE5048272 | Probe | FAM | 134 | hg19\|chr1:13936907-13940198:+  CAGGCATTCGCATCGAGGATCTGCCAACTTCAGAAAGCACAGTCCACGCGCAAGAACAAAGTCCAAGCGCCACAGCCTCAAACGTGGCCACCAGTCACTCCACGGAGAAAGTGGATGGAGAC |
| PHEX | dHsaCPE5033810 | Probe | FAM | 90 | hg19\|chrX:22265986-22266107:+  TAACTTTGAAGAATTCCAGAAAGCTTTTAACTGTCCACCCAATTCCACGATGAACAGAGGCATGGACTCCTGCCGACTCTGGTAGCTGGGACGCTGGTTTATGGCATCCTGAGACAGTTGCA |
| COL1A1 | dHsaCPE5034390 | Probe | FAM | 113 | hg19\|chr17:48277176-48277297:+  TTGGTCTCGTCACAGATCACGTCATCGCACAACACCTTGCCGTTGTCGCAGACGCAGATCCGGCAGGGCTCGGGTTTCCACACGTCTCGGTCATGGTACCTGAGGCCGTTCTGTACGCAGGT |
| BGLAP | dHsaCPE5031314 | Probe | FAM | 69 | hg19\|chr1:156211947-156212069:+  TATAAACAGTGCTGGAGGCTGGCGGGGCAGGCCAGCTGAGTCCTGAGCAGCAGCCCAGCGCAGCCACCGAGACACCATGAGAGCCCTCACACTCCTCGCCCTATTGGCCCTGGCCGCACTTTG |
| SPP1 | dHsaCPE5045616 | Probe | FAM | 66 | hg19\|chr4:88902913-88903727:+  GGTCAAAATCTAAGAAGTTTCGCAGACCTGACATCCAGTACCCTGATGCTACAGACGAGGACATCACCTCACACATGGAAAGCGAGGAGTTGAATGGTGCATACAAGGCCATCCCCGTTGCC |
| ALPL | dHsaCPE5039244 | Probe | FAM | 61 | hg19\|chr1:21902282-21902404:+  GCGGTGGAGATGGACCGGGCCATCGGGCAGGCAGGCAGCTTGACCTCCTCGGAAGACACTCTGACCGTGGTCACTGCGGACCATTCCCACGTCTTCACATTTGGTGGATACACCCCCCGTGGC |
| OPG | dHsaCPE5039722 | Probe | FAM | 84 | hg19\|chr8:119936545-119936666:+  TGAGAAACAGTTTACTCATCCATGGGATCTCGCCAATTGTGAGGAAACAGCTCAATGGCCATTTCCAGTTATAAGCAGCTTATTTTTACTGATTGGACCTGGTTACCTATCATTTCTAAAAA |
| CSF1 | dHsaCPE5042260 | Probe | FAM | 69 | hg19\|chr1:110458294-110460012:+  TGAGTTTGTAGACCAGGAACAGTTGAAAGATCCAGTGTGCTACCTTAAGAAGGCATTTCTCCTGGTACAAGACATAATGGAGGACACCATGCGCTTCAGAGATAACACCCCCAATGCCATCGC |
| EPHB4 | dHsaCPE5043006 | Probe | FAM | 80 | hg19\|chr7:100400884-100401005:+  TCCCCGAGGTGGCTGGGGGGTGATTTTCCCCTCCTATTATGGCAGAACCCCCAAATCCTGTCTCTCCAAATTGCCAACTCCTCACCCCACGGGCTCAAAGTGCAATCCAGCGGGGCACAGGG |
| SEMA3A | dHsaCPE5031396 | Probe | FAM | 64 | hg19\|chr7:83823879-83824000:+  GACAATCCTAGTTAACCAGCCCATGCTGCAGACGCTGTAGGTCCCTTTGCTGCTTTAGTCTTCCTTCCTGTATTGTGCGGCCAGAGAAGTTCAAACAATCTGGAAACTGGAGGTAACAGGTG |
| VDR | dHsaCPE5058214 | Probe | FAM | 147 | hg19\|chr12:48251447-48258933:+  TGCACTTCCTCATCTGTCAGAATGAACTCCTTCATCATGCCGATGTCCACACAGCGTTTGAGCCGGCAGGCCTGGCAGTGGCGTCGGTTGTCCTTGGTGATGCGGCAGTCCCCGTTGAAGGGGCAGGTG |
| HPRT1 | dHsaCPE5192872 | Probe | HEX | 90 | hg19\|chrX:133627550-133632642:+  ACTGGCAAAACAATGCAGACTTTGCTTTCCTTGGTCAGGCAGTATAATCCAAAGATGGTCAAGGTCGCAAGCTTGCTGGTGAAAAGGACCCCACGAAGTGTTGGATATAAGCCAGACTTTGTT |
| HMBS | dHsaCPE5193438 | Probe | HEX | 137 | hg19\|chr11:118960941-118962210:+  AGAGAAAGTTCCCGCATCTGGAGTTCAGGAGTATTCGGGGAAACCTCAACACCCGGCTTCGGAAGCTGGACGAGCAGCAGGAGTTCAGTGCCATCATCCTGGCAACAGCTGGCCTGCAGCGCA |

## 6 Reference gene expression and effect of the normalization of examined genes to an internal reference gene

**
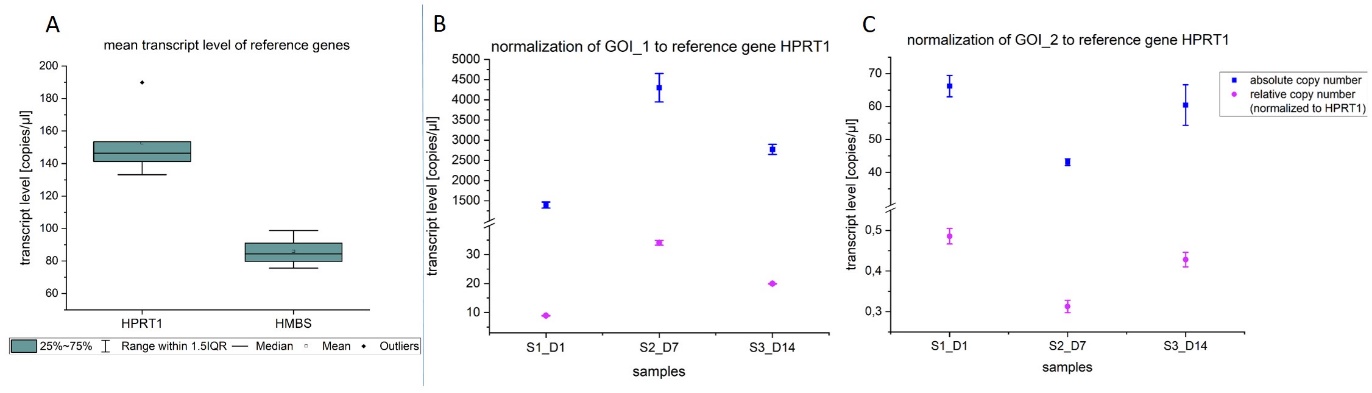
**

**Supplementary Figure S4. (A)** Evaluation of the stable gene expression of HPRT1 (Hypoxanthine phosphoribosyltransferase 1) and HMBS (Hydroxymethylbilane synthase ) in primary human osteoblasts under different culture conditions. The cDNA load corresponded to 10 ng RNA input per 20 µl PCR reaction. The samples were derived from cells cultured in 2D monolayer and 3D-microchip culture conditions, and for different time periods (each sample 8-12 ddPCR replicates). Figure shows the mean ± SD (n=5). **(B** and **C)** Representative transcript levels of selected genes of interest (GOI) with high **(B)** and low **(C)** copy concentration in one culture configuration at day 1, 7 and 14. The cDNA load corresponded to 10 ng RNA input per 20 µl PCR reaction. The transcript levels before and after normalization to the internal reference gene (duplex ddPCR) are presented as absolute copy number (blue) and relative copy number (pink) per µl ddPCR reaction (20 µl total ddPCR reaction), respectively. Figures show the mean ± SD of two ddPCR replicates (n=2). (GOI = gene of interest)

**7 Relative gene expression of biomarkers differently and not differently expressed between osteoblast derived from alveolar bone (hOB-A) and iliac crest (hOB-IC) of the same donor**

**
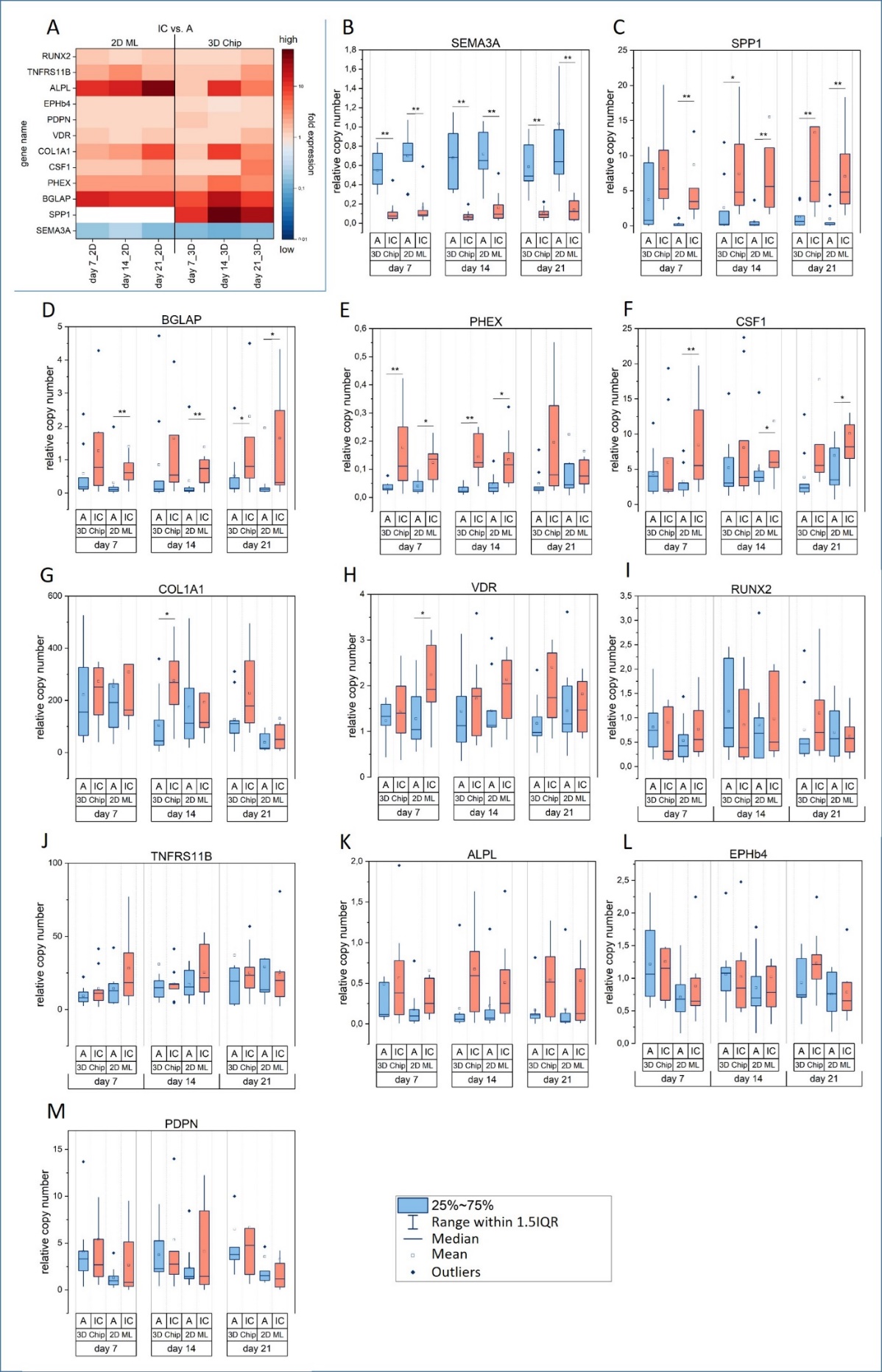
**

**Supplementary Figure S5.** hOB were cultured in 3D-microchip and 2D ML culture under osteogenic conditions. The copy numbers of the target genes at days 7, 14 and 21 were normalized to copy numbers of the reference gene HPRT1 (hypoxanthine phosphoribosyl-transferase 1). *p< 0.05 **p<0.01 for comparison of hOB-A (blue) with hOB-IC (red); Wilcoxon signed-rank test, n = 9 donors. SEMA3A (semaphorin-3A), SPP1 (osteopontin), BGLAP (osteocalcin), PHEX (phosphate regulating endopeptidase, X-linked), CSF1 (colony stimulating factor 1), COL1A1 (collagen type 1 alpha 1 chain) and VDR (vitamin D receptor). The expression differences between hOB-A and hOB-IC were not significant for RUNX2 (runt related transcription factor 2), TNFRSF11 (TNF receptor superfamily member 11b), ALPL (alkaline phosphatase, liver/bone/kidney), EPHB4 (ephrin receptor B4) and PDPN (podoplanin).

**8 Relative gene expression of biomarkers differently and not differently expressed between osteoblast in 3D-microchip and 2D ML culture and/or by culture time**

**
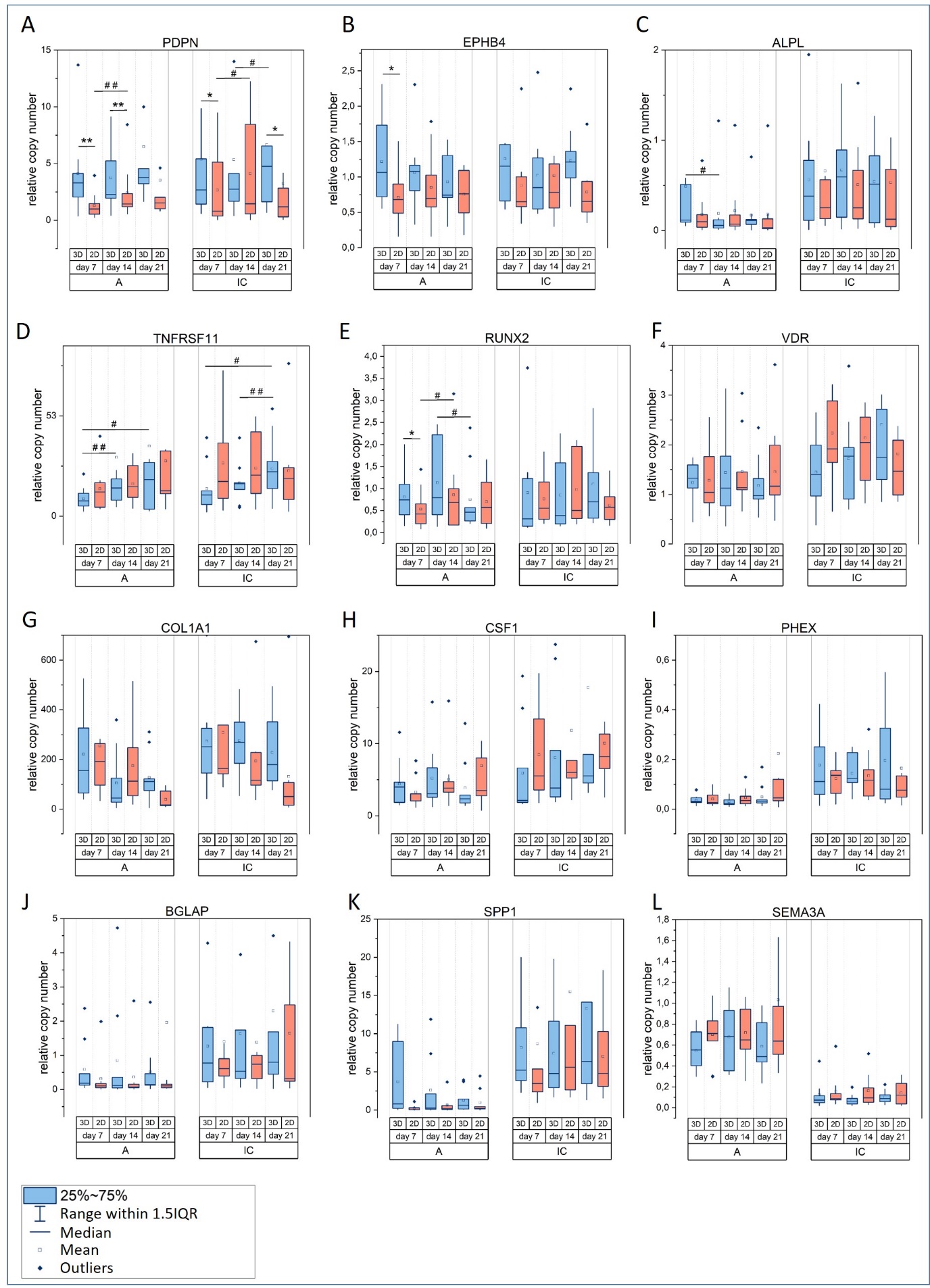
**

**Supplementary Figure S6.** hOB were cultured in 3D-microchip and 2D ML culture under osteogenic conditions. Color code of the comparison groups: 3D-microchip in blue and 2D ML in red. The copy numbers of the target genes at days 7, 14 and 21 were normalized to copy numbers of the reference gene HPRT1 (hypoxanthine phosphoribosyl-transferase 1). *p< 0.05, **p<0.01 for comparison of 3D-microchip with 2D ML culture; ^#^p< 0.05, ^##^p<0.01 for comparison of culture time; Wilcoxon signed-rank test, n = 9 donors. PDPN (podoplanin), EPHB4 (ephrin receptor B4), ALPL (alkaline phosphatase, liver/bone/kidney), TNFRSF11 (TNF receptor superfamily member 11b), RUNX2 (runt related transcription factor 2). The expression differences between hOB-A and hOB-IC were not significant for VDR (vitamin D receptor), COL1A1 (collagen type 1 alpha 1 chain), CSF1 (colony stimulating factor 1), PHEX (phosphate regulating endopeptidase, X-linked), BGLAP (osteocalcin), SPP1 (osteopontin), SEMA3A (semaphorin-3A)

**9 Relative gene expression of biomarkers differently and not differently expressed between osteoblast derived from alveolar bone (hOB-A) and iliac crest (hOB-IC) of the same donor after 1,25D3 treatment**


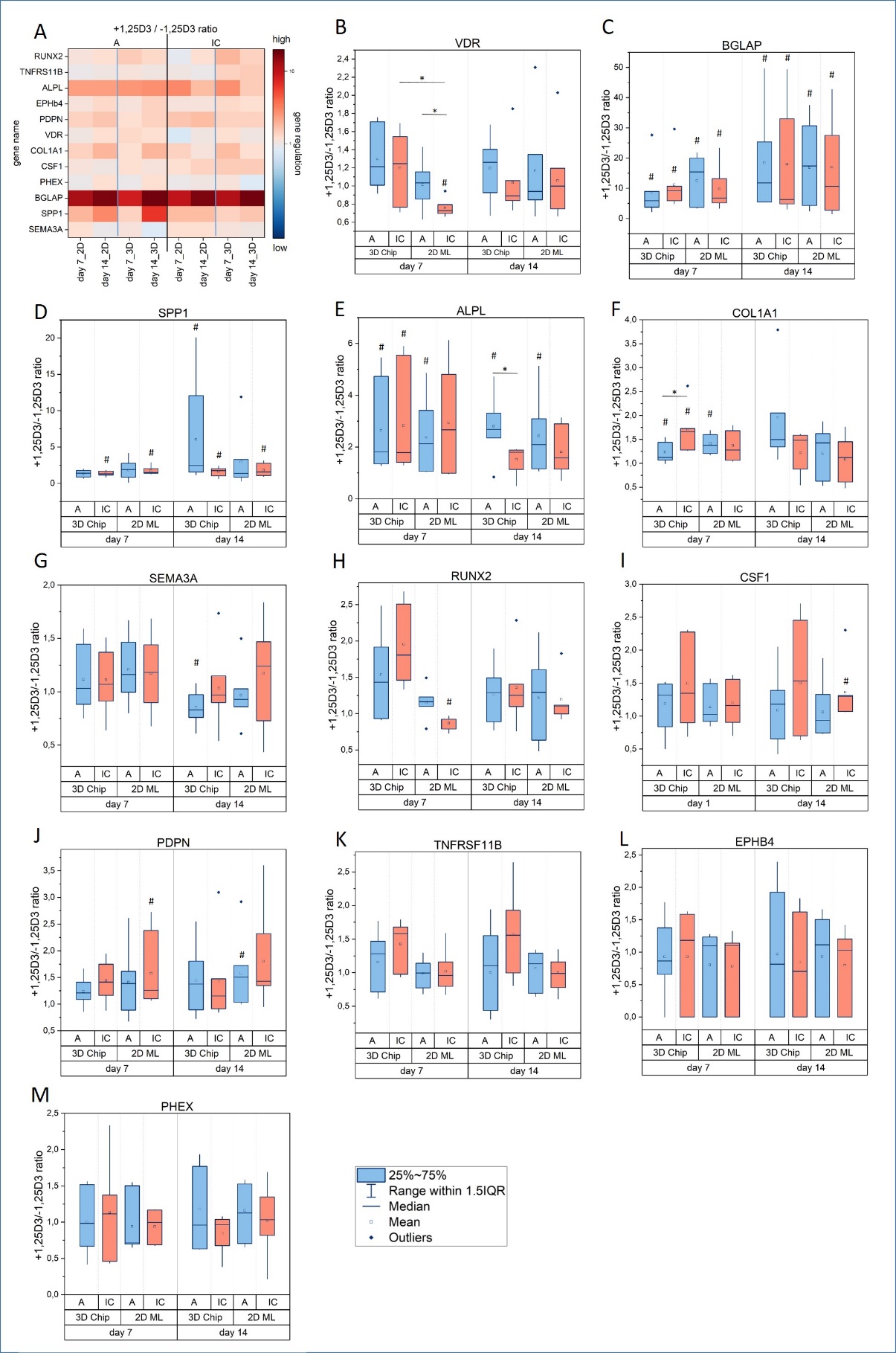


**Supplementary Figure S7.** Effect of 1,25D3 treatment on gene expression in osteoblasts derived from alveolar bone (hOB-A, blue) and iliac crest (hOB-IC, red). The cells were cultured in 3D-microchip and 2D ML culture under osteogenic conditions for 7 and 14 days with or without (+/-) addition of 1,25D3 (1,25-dihydroxyvitamin D3). The copy numbers of the target genes were normalized to copy numbers of the reference gene HPRT1 (hypoxanthine phosphoribosyl-transferase 1). The effect of 1,25D3 treatment is given by the +1,25D3/-1,25D3 ratio of normalized copy numbers and describes the fold expression of biomarkers in 1,25D3-treated versus untreated cell cultures. A ratio of 1 means no regulation, >1 up-regulation, and <1 down-regulation. ^#^p< 0.05 for comparison of 1,25D3 treated with non-treated cultures (+1,25D3 vs. -1,25D3); *p< 0.05 for comparison of the 1,25D3 effect between culture modes, i.e. culture configuration, time and cell origin; Wilcoxon signed-rank test, n = 7 donors; n = 5 donors for COL1A1. The expression differences between hOB-A and hOB-IC were not significant for TNFRSF11B (TNF receptor superfamily member 11b), EPHB4 (ephrin receptor B4) and PHEX (phosphate regulating endopeptidase, X-linked).
